# Characteristics of human encounters and social mixing patterns relevant to infectious diseases spread by close contact: a survey in Southwest Uganda

**DOI:** 10.1186/s12879-018-3073-1

**Published:** 2018-04-11

**Authors:** O. le Polain de Waroux, S. Cohuet, D. Ndazima, A. J. Kucharski, A. Juan-Giner, S. Flasche, E. Tumwesigye, R. Arinaitwe, J. Mwanga-Amumpaire, Y. Boum, F. Nackers, F. Checchi, R. F. Grais, W. J. Edmunds

**Affiliations:** 1 Department of Infectious Disease Epidemiology, London School of Hygiene and Tropical Medicine, London, UK; 2 Epicentre, Paris, France; 3 Epicentre, Uganda Research Centre, Mbarara, Uganda; 4 Mbarara University Of Science and Technology (MUST), Mbarara, Uganda; 5 Kabwohe Medical Research Centre, Kabwohe, Uganda; 6 Epicentre, Brussels, Belgium

**Keywords:** Social contact, Infectious diseases, Close contact transmission, Uganda, Survey

## Abstract

**Background:**

Quantification of human interactions relevant to infectious disease transmission through social contact is central to predict disease dynamics, yet data from low-resource settings remain scarce.

**Methods:**

We undertook a social contact survey in rural Uganda, whereby participants were asked to recall details about the frequency, type, and socio-demographic characteristics of any conversational encounter that lasted for ≥5 min (henceforth defined as ‘contacts’) during the previous day. An estimate of the number of ‘casual contacts’ (i.e. < 5 min) was also obtained.

**Results:**

In total, 566 individuals were included in the study. On average participants reported having routine contact with 7.2 individuals (range 1-25). Children aged 5-14 years had the highest frequency of contacts and the elderly (≥65 years) the fewest (*P* < 0.001). A strong age-assortative pattern was seen, particularly outside the household and increasingly so for contacts occurring further away from home. Adults aged 25-64 years tended to travel more often and further than others, and males travelled more frequently than females.

**Conclusion:**

Our study provides detailed information on contact patterns and their spatial characteristics in an African setting. It therefore fills an important knowledge gap that will help more accurately predict transmission dynamics and the impact of control strategies in such areas.

**Electronic supplementary material:**

The online version of this article (10.1186/s12879-018-3073-1) contains supplementary material, which is available to authorized users.

## Background

Quantification of human interactions relevant to the spread of infectious diseases transmitted by close contact is essential to accurately predict their infection dynamics and better predict the impact of control strategies [1, 2]. Detailed studies of social mixing patterns have now been undertaken in a number of settings [2–12]. Those studies have shown that people tend to mix with other individuals of their own age (i.e. assortative mixing); however, the frequency of contact, the degree of intergenerational mixing and the characteristics of mixing tend to vary between settings, depending on factors such as household size, population density and local activities, among others [3–11, 13].

Importantly, while the burden of infectious diseases remains disproportionally high in low-income settings, social contact data to help improve our understanding of infectious disease dynamics in such settings remain scarce. Three studies in Africa have been published to date, including in Kenya, South Africa, and in Zimbabwe [10, 12, 13], but no study has been undertaken in Uganda. Evidence from the current studies show strong age-assortativity in young age groups (children tend to interact proportionally more with children in their age group than with others), but also important intergenerational mixing, more so than seen in European or other high-income settings [4].

In addition, with the exception of a recent study from China [11], the spatial dispersal of social contacts relevant for transmission has often been overlooked, and there is – to our knowledge – no published information from low-income settings on the spatial characteristics of social contacts. Spatial mobility is particularly important for epidemic risk prediction of novel and re-emergent diseases, and for the optimization of routine control programmes [14].

To address this knowledge gap, we set up a study of social contacts relevant to the spread of infections transmitted through the respiratory route or by close contact, in rural southwest Uganda.

## Methods

The study was conducted in four sub-counties of Sheema North Sub-District (southwest Uganda), an area with a total of about 80,000 inhabitants. About half (49%) of the district’s population is < 15 years. The area is primarily rural.

### Study design

Between January and March 2014 survey teams undertook interviews of a subset of individuals who were also included in a survey of *Streptococcus pneumoniae* carriage [15], asking about their social contacts in the 24 h preceding the survey, including the frequency, type and duration of encounters.

Our target sample size was 687, including all 327 individuals aged ≥15 years included in the nasopharyngeal carriage study within which this study was nested [15] and a subsample of 90 children in each of the following age groups: < 2 year olds, 2 – 4 years old, 5 – 9 years and 10 – 14 years old. Based on estimates from previous findings available at the time [12, 16], such sample size provided a precision of just over 1 contact on the mean number of contacts per day, and enabled detection of a 20% difference in the average number of daily contacts by age group.

Individuals were selected from 60 clusters randomly sampled from the 215 villages and two small towns in the sub-county, with an inclusion probability proportional to the size of the village or town. Within each cluster 11 or 12 households were selected at random from a list of households. A household was defined as a group of individuals living under the same roof and sharing the same kitchen on a daily basis. One individual from each household was randomly selected from a list of predefined age groups to sample from within each cluster. When nobody in the household was from that age group, either someone from another age group was selected providing that the quota for that age group had not been reached in the cluster, or the closest neighbouring household was visited instead. In case of non-response, another attempt was made later in the day or the following Saturday. After the second attempt, the individuals were not replaced.

### Data collection

Informed consent was sought for individuals aged > 17 years, and from a parent or carer for children < 18 years. In addition, assent was sought from children aged 7 – 17 years. Participants were asked to recall information on the frequency, type and duration of social encounters from the time they woke up the day before the survey until when they woke up on the survey day (~ 24 h).

We defined contacts as two-way conversational encounters lasting for ≥5 min. Participants were first asked to list all the places they had visited in the previous 24 h, the number of people they had contact with, their relationship with each individual mentioned, the age (or estimated age) of each listed contact and how long the encounter lasted for. Contacts involving skin-to-skin touch or sharing utensils passed directly from mouth-to-mouth were defined as ‘physical’ contacts. The questionnaire can be found in the Additional file 1.

We defined short contacts lasting less than 5 min as ‘casual contacts’. Participants were only asked to estimate the number of casual contacts they had, based on pre-defined categories (< 10, 10-19, 20-29, ≥30), but were not asked to provide detailed information about the nature of the encounter or the socio-demographic characteristics of the person met. Casual contacts are generally inaccurately reported in social contact surveys [7], particularly in a retrospective design, and most contacts important for the transmission of respiratory infections are believed to be close rather than casual [6].

The questionnaire was designed in English, translated to Ruyankole, the local language, and back-translated to English for consistency. For children < 5 years, parents were asked about their child’s encounters and whereabouts. Children aged 5 – 14 years were interviewed directly, using a questionnaire with a slightly adapted wording from that used for adults.

Geographical coordinates from each participant’s household and the centre of each village were taken using handheld GPS devices. The spatial identification of each location in the area was done by the research team during the preparation phase of the study. Geo-referencing of each village, hamlet or town in the area, was done using GIS imagery as well as by travelling to the different villages to collect that information using handheld GPS devices. Given that some villages had very similar names, interviewers carried with them a list of all of those (> 300), so as to avoid data entry problems.

Questionnaires completed in the field were double entered on a preformatted data entry tool (www2.voozanoo.net, Epiconcept, France) by two data managers working independently. Data entry conflicts were identified automatically and resolved as the data entry progressed.

### Ethics

Approval was obtained from the Ethical review boards of Médecins Sans Frontières (MSF), the Faculty of Medicine Research & Ethics Committee of the Mbarara University of Science and Technology (MUST), the Institutional Ethical Review Board of the MUST, the Uganda National Council for Science and Technology (UNCST) and the London School of Hygiene and Tropical Medicine (LSHTM).

### Analysis

#### Characteristics of social contacts by time, person and place

We analysed the frequency distribution of contacts for a set of covariates, including age, sex, and occupation, day of the week, distance travelled, and type of contact. Encounters reported with the same individual in different settings counted as one contact only. Straight-line distances between the centre point of all villages and towns in the dataset were calculated, and these were then used to evaluate how far people travelled, based on the reported names of villages and town where each reported encounter took place, and their own village or town of residence.

We used negative binomial regression to estimate the ratio of the mean contacts as a function of the different covariates of interest. Negative binomial was preferred over Poisson regression given evidence of over-dispersion (variance > mean, and likelihood ratio significant (*P* < 0.05) for the over-dispersion parameter). We considered variables associated with contact frequency at *p* < 0.10 for multivariable analysis, and retained them in multivariable models if they resulted in a reduction of the Bayesian Information Criterion (BIC).

Next, we explored whether people reporting a high frequency of casual contacts (≥10 casual contacts) differed from those reporting fewer contacts with regards to their socio-demographic characteristics. We did so using log-binomial regression to compute crude and adjusted relative risks (RRs) for having a high frequency. In all analyses we accounted for possible within-cluster correlation by using linearized based variance estimators [17]. Analyses were also weighted for the unequal probabilities of sampling selection by age group.

#### Age-specific social contact patterns

We analysed the age-specific contact patterns through matrices of the mean number of contacts between participants of age group *j* and individuals in age group *i*, adjusting for reciprocity, as in Melegaro et al. [6].

If *x*_*ij*_ denotes the total number of contacts in age group *i* reported by individuals in age groups *j*, the mean number of reported contacts (*m*_*ij*_) is calculated as *x*_*ij*_/*p*_*j*_, where *p*_*j*_ is the study population size of age group *j*. At the population level the frequency of contacts made between age groups should be equivalent such that *m*_*ij*_*P*_*j*_ = *m*_*ji*_*P*_*i*_. The expected number of contacts between the two groups is thereforeC_*ij*_= (*m*_*ij*_*P*_*j*_ + *m*_*ji*_*P*_*i*_)/2. Hence, the mean number of contacts corrected for reciprocity $\left({m}_{ij}^C\right)$ can be expressed as *C*_*ij*_/*P*_*j*_.

We tested the null hypothesis of proportionate mixing by computing the ratio of observed mixing patterns to that of expected mixing patterns if social contact occurred at random. Under the assumption of random mixing, the probability of encounter between age groups thus depends on the population distribution in each age group, and the contact matrix under this random mixing hypothesis was calculated based on the percentage of population in each age group. The ratio of observed over expected contacts was then computed, and confidence intervals were obtained through bootstrapping, with replacement, for a total of 1000 iterations. This approach is similar to that taken by others [11].

#### Epidemic simulations

Finally, in order to explore the infection transmission dynamics resulting from our contact pattern data, we simulated the spread of an immunizing respiratory infection transmitted through close contact in a totally susceptible population, thus assuming a Susceptible-Infected-Recovered (SIR) model. The model contained nine mixing age groups, with a transmission rate *β*_*ij*_ at which individuals in age group *j*come into routine contact with individuals in age group *i* computed as ${\beta}_{ij}={qm}_{ij}^C/{\omega}_i$, where *ω*_*i*_ is the proportion of individuals in age group *i*, and ${qm}_{ij}^C$ is the next generation matrix, with *q*representing the probability of successful transmission per contact event [18]. We assumed *q* to be homogeneous and constant across all age groups and conducted a set of simulations for fixed values of *q* between 25% and 40%, in line with what has been reported with influenza pandemic strains [18, 19]. The basic reproduction number (*R*_0_) – which corresponds to the average number of people infected by one infectious individual in a totally susceptible population – was calculated as the dominant eigenvalue of the next generation matrix. We took uncertainty estimates in the contact matrices (and hence final size outputs) into account by iterating the model on bootstrapped matrices.

We did not develop a model to run simulations to model the dynamic of the epidemic in each setting. Rather, we computed the final epidemic size (i.e. the number of individuals who would have been infected during the epidemic) for each specific age group, based on simple a mass action model adapted to account for multiple age classes, as described in Kucharski et al. [20], with the following equation:

$$ {F}_i=1-\exp \left(-\sum \limits_{j=1}^9{\beta}_{ij}{\omega}_j{F}_j\right) $$

,where *F* represents the final epidemic size by age group (i.e. the proportion of individuals who are infected in each age group).

Estimates obtained using the contact data from Uganda were compared to that of Great Britain, using data from the POLYMOD study [4] for the latter and a similar approach to compute the mixing matrix. The model was parameterised with social contact data on physical contacts only, lasting ≥5 min, rather than all contacts, given that physical contacts generally seem to better capture contact structures relevant for the transmission of respiratory infections [6]. Data for Great Britain were available for the same age groups, and for physical contacts specifically, in the same way that physical contacts were defined in our study, which made the data for physical contacts only more comparable between studies than that of overall contacts.

Analyses were performed in either Stata13.1 IC or R version 3.2 [21].

## Results

### Study population

A total of 566 individuals participated in the survey. This corresponded to an overall response rate of 83%, higher among ≥15 years old (98%), and lower among under 2 s (68%), 2-4 year olds (64%), 5 – 9y olds (82%) and 10 - 14y olds (57%). There were more female (58%) than male respondents, but this differed by age group, with fewer females in young age groups and more adult females than males (Table S1 in Additional file 2).

The mean household size was 5.3 (median 5, range 1 – 18). Almost all (98%) school-aged children aged 6 – 14 years attended school or college. Among adults, agriculture was the main occupation and about 27% of the females were homemakers/housewives (Table 1).

**Table 1:** Mean Number of Reported Contacts and Ratio of Means By Socio-demographic Characteristic of The Study Population, Sheema, Uganda, January – March 2014

Variables	Number	Mean number of contacts (95%CI)	Crude RoM (95%CI)	Age adjusted RoM (95%CI)
Age groups				
< 2y	61	6.11 (5.42,6.81)	0.99 (0.83,1.17)	
2-4y	57	6.70 (6.08,6.81)	1.08 (0.95,1.23)	
5-9y	74	8.50 (7.79,9.20)	1.37 (1.18,1.59)	
10-14y	51	8.70 (7.52,9.90)	1.40 (1.19,1.66)	
15-24y	91	6.20 (5.51,6.88)	ref	
25-34y	57	6.89 (5.99,7.80)	1.11 (0.93,1.33)	
35-44y	55	7.73 (6.99,8.46)	1.25 (1.09,1.43)	
45-54y	46	7.74 (6.37,9.11)	1.25 (1.01,1.54)	
55-64y	26	6.27 (5.01,7.53)	1.01 (0.80,1.29)	
65 + y	48	4.85 (4.18,5.53)	0.78 (0.64,0.95)	
Sex				
Female	330	7.05 (6.66,7.44)	ref	
Male	236	7.47 (6.82,8.11)	1.06 (0.96,1.18)	
Occupation/daily activity				
Pre-school child	93	7.00 (6.30,7.70)	1.06 (0.90,1.23)	1.26 (1.02,1.55)
Student	166	8.27 (7.56,8.98)	1.27 (1.10,1.46)	1.30 (1.05,1.63)
Office worker	4	11.34 (9.64,13.03)	1.81 (1.20,2.73)	1.70 (1.32,2.18)
Shop worker	34	6.82 (5.56,8.08)	1.03 (0.84,1.25)	1.03 (0.83,1.29)
Agriculture	106	7.30 (6.58,8.01)	1.12 (0.97,1.30)	1.12 (0.96,1.30)
Other manual worker	40	5.37 (4.36,6.38)	0.85 (0.70,1.04)	0.85 (0.68,1.06)
At home	60	6.43 (5.62,7.24)	ref	ref
Unemployed	11	6.44 (2.92,9.96)	0.83 (0.60,1.15)	1.22 (0.77,1.94)
Retired	8	4.77 (3.75,5.80)	0.71 (0.48,1.05)	0.89 (0.70,1.13)
Other/unreported	41	6.65 (5.80,7.51)	1.02 (0.85,1.23)	1.18 (0.98,1.43)
Don’t know	1	–	–	–
Day of the week				
Weekday	441	7.14 (6.79,7.49)	ref	
Sunday	125	7.50 (6.56,8.44)	1.05 (0.92,1.20)	1.04 (0.92,1.18)
Travel outside village/town in previous 24 hours				
No	427	6.57 (6.20,6.92)	ref	
Yes	139	9.04 (8.35,9.73)	1.38 (1.25,1.52)	1.35 (1.22,1.49)
Number of casual contacts				
< 10	315	5 (1 -15)	Ref	Ref
10 -19	119	8 (2-23)	1.43 (1.28, 1.59)	1.39 (1.25, 1.55)
≥ 20	56	9 (2-25)	1.64 (1.44; 1.86)	1.61 (1.43,1.83)
Don’t know	76	8 (0-19)	1.52 (1.37; 1.68)	1.45 (1.29, 1.64)

*CI* Confidence interval, *RoM* ratio of means

### Characteristics of contacts

#### Contacts (i.E. ≥5 min long)

**Fig. 1:**
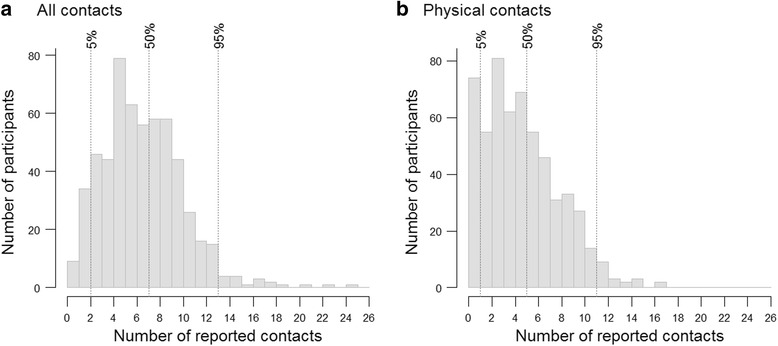
Number of Reported Contacts, Including All Contacts (**a**) and Physical contacts (**b**), Sheema, Uganda, January – March 2014. the vertical dotted lines represent the 5% centile, the median and 95% centile of the total number of reported contacts. The x axis ticks are placed on the left side of the bars

A total of 3965 contacts with different individuals were reported, corresponding to an average of 7.2 contacts per person (median 7, range 0 - 25) (Fig. 1). The majority of contacts were physical, thus involving skin-to-skin contact (mean 5.1, median 5 (range 0 – 18)). The age and sex distribution of study participants can be found in Table S1 (Additional file 2 with supplementary figures and tables).

Over half of all contacts (*n* = 2060 (52%)) were with household members, 627 (16%) with other relatives, 873 (22%) with colleagues/friends/schoolmates and 402 (10%) with other individuals. The duration of routine contacts is shown in Figure S1 (Additional file 2).

Most contacts (82%) were with individuals who would be normally seen daily, 520 (13%) with people normally seen at least weekly, 4% with people met more rarely and 1% of the reported contacts were with people that the participants had never met before.

We found marked differences in the number of contacts by age group, but not by sex. School-aged children reported the highest daily number of contacts, while the elderly had the fewest (Table 1). There was no difference in the mean number of contacts for individuals living in the district towns of Kabwohe and Itendero (*n* = 43) and the 523 others living in surrounding villages (chi-square test *P*-value = 0.79). Table 1 provides further details about the population characteristics, the mean number of contacts by socio-demographic and other covariates, as well as the ratio of mean contacts by covariate. Age was the only confounding factor.

**Fig. 2:**
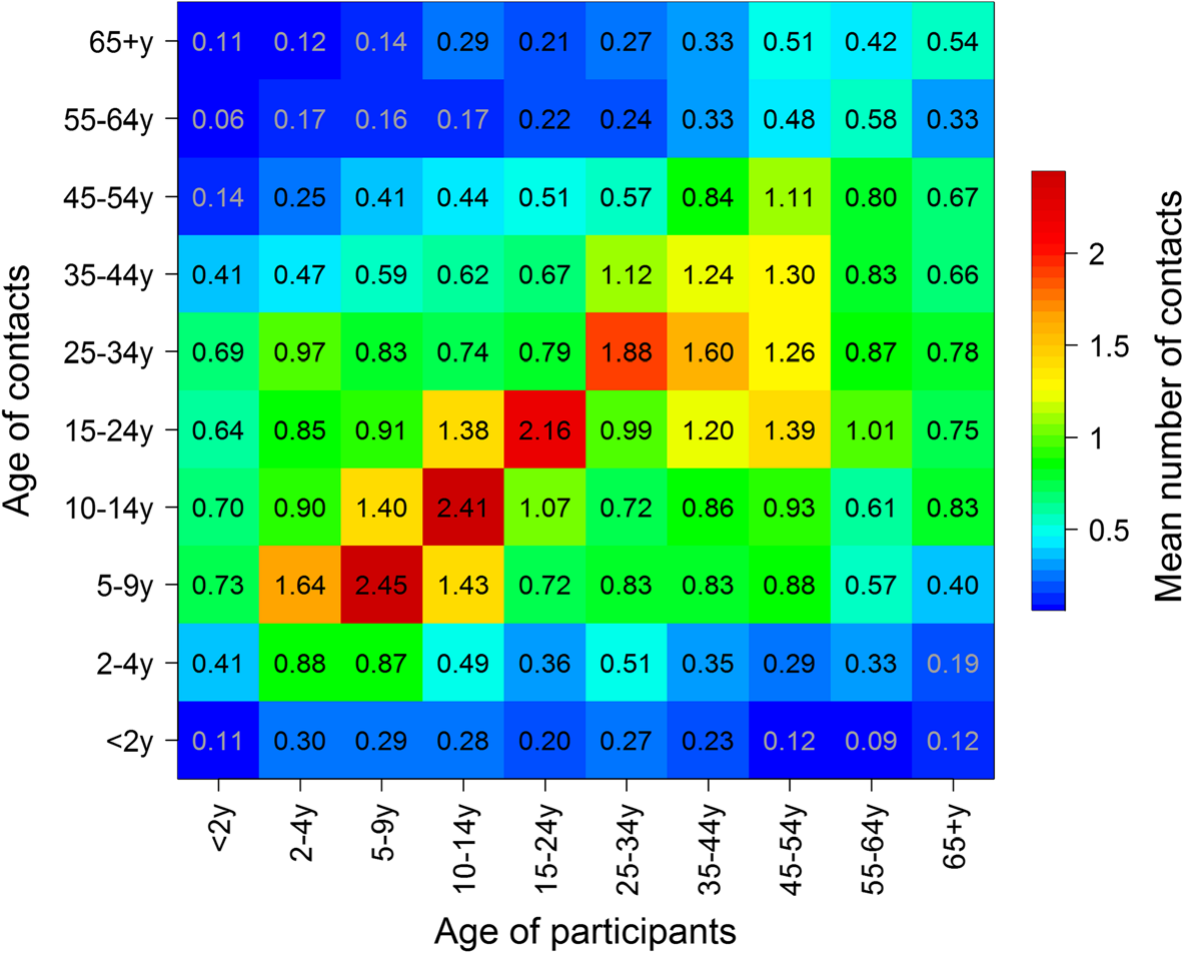
Average Number of Reported Contacts By Age Group, Sheema, Uganda, January – March 2014. Numbersin each cell represent the average number of contacts between between age groups corrected for reciprocity

**Fig. 3:**
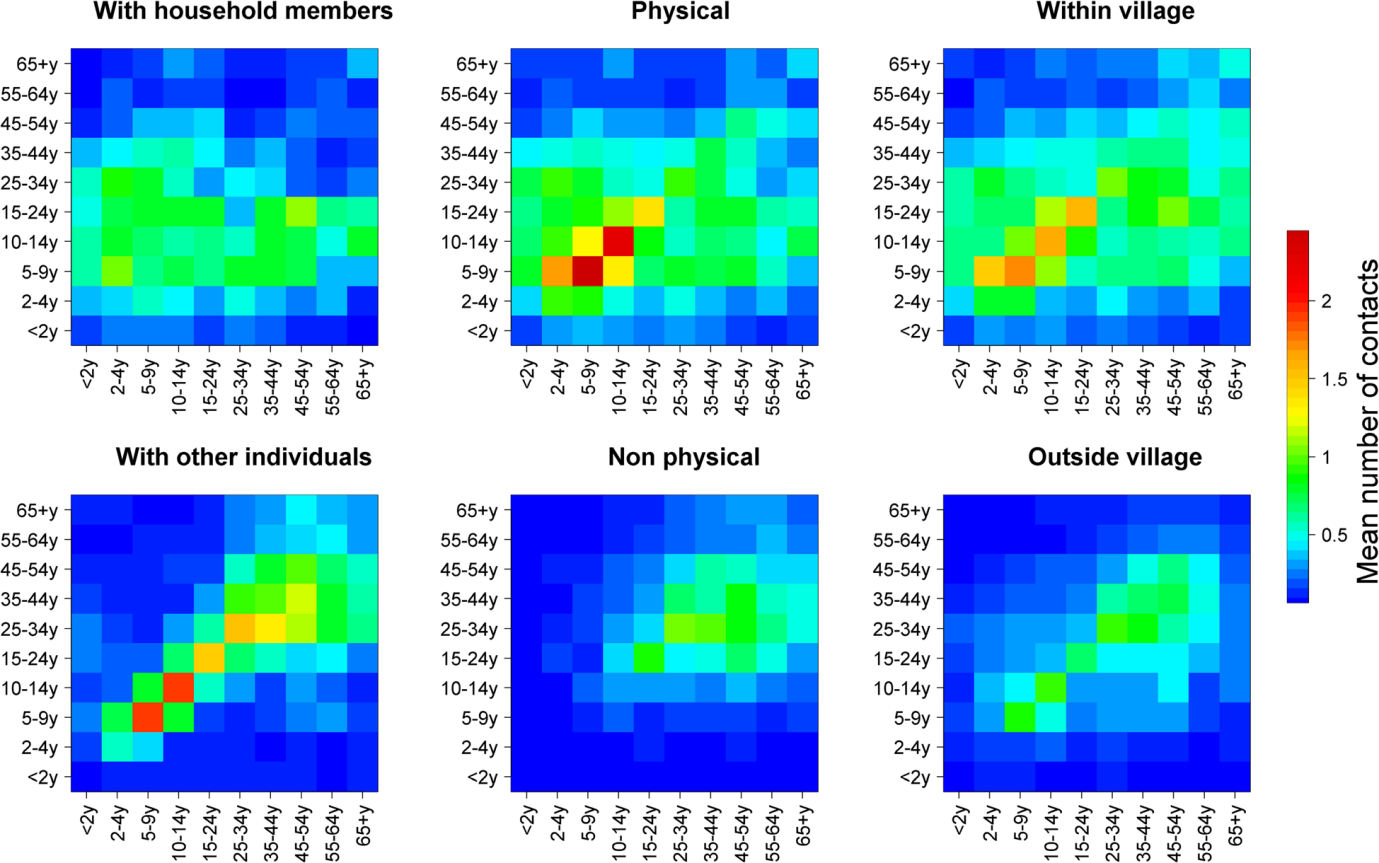
Contact Matrices With Household members and Non-Household Members (Left upper and lower panel), for Physical and Non-Physical Contacts (Middle upper and lower panel), and for Contacts Made Within and Outside the Village (Right upper and lower panel), Sheema, Uganda, January – March 2014

Overall, contacts tended to be assortative, as shown by the strong diagonal feature on Fig. 2, with most of the intergenerational mixing occurring within households (Fig. 3). Only teenagers and adults reported non-physical contacts (Fig. 3). The quantification of assortativity can be seen in Figures S2a-c (Additional file 2), which show the ratios of observed contacts, as obtained in the survey but corrected for reciprocity, to that of expected contacts under the proportionality assumption, for all contacts and physical contacts only. The results show age-assortativity of contacts, for all age groups (other than < 2 year olds) for all contacts, and primarily for school-aged children when considering physical contacts only.

Reciprocity correction accounted for differences in reporting of contacts by age groups, particularly a proportionally higher frequency of contacts reported by young children with older age groups than older age groups reported with young children, as shown in Figure S3 (Additional file 2).

There was no statistical difference in the average number of contacts between weekend (Sunday) and weekdays (Monday, Tuesday, Thursday and Friday) (Table 1). As shown in Table 1, mean number of reported contacts on Sundays was 7.50 (95%CI 6.56; 8.44), slightly higher on average than on weekdays, where the average was 7.14 (6.79; 7.49), which was not statistically significant (*P* = 0.229). The balance of respondents reporting contacts from weekdays and weekends reflected the normal proportion of week vs. weekend days in a normal week.

**Fig. 4:**
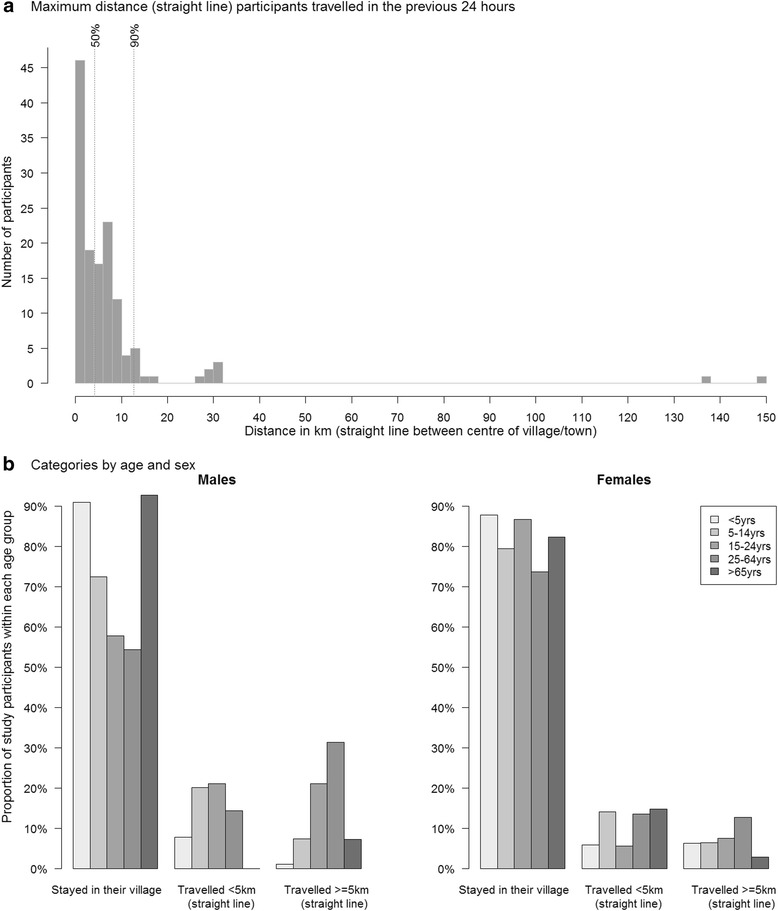
Distance Travelled By Study Participants in the 24 Hours Preceding the Survey, Overall (**a**) and By Categories of Distance, Age and Sex (**b**), Sheema, Uganda, January – March 2014

About a quarter (*n* = 136 (24%)) of participants reported social encounters outside their village of residence, and about 12% of contacts occurred outside participants’ village of residence. The majority (56%) of people who travelled outside their village went to places located within a 5 km radius from the centre point of their village of residence, and 90% stayed within 12 km (Fig. 4). Adult males tended to travel more than females (Fig. 4). Overall, 29% of males had contact with someone outside their village, compared to 20% females (χ^2^, *P* = 0.0406). Most (87%) children under 5 years of age stayed in their village, whereas about a quarter or more individuals travelled outside their village among 5 – 14 years old (25%), 15-44 years old (32%) and ≥ 45 years of age (25%), a difference by age group which was significantly different (*P* = 0.0081).

Overall, 30% of males travelled outside their village compared to 20% of females. When stratifying by age, the difference between sex were more marked, with no statistical difference between males and females < 5 years of age (*P* = 0.296) or 5 – 14 year olds (*P* = 0.272), but marked differences among adults (≥15 years old), with 42% of males travelling outside of their village compared to 24% of females (*P* = 0.0037).

The proportion of individuals who travelled outside their village differed by occupation (although not statistically significantly, with shop keepers (38%), those working in agriculture (31%) and office workers (50%) travelling outside their village more than others.

Most contacts made outside the household as well as those with individuals outside participants’ village were mostly assortative (Fig. 3), and the proportion of contacts outside the village was different by age group (*P* < 0.001); higher among adults, increasingly so as distance from home increased (Fig. 4).

#### ‘Casual’ contacts (< 5 min long)

Information on the number of casual contacts was reported by 490 (87%) participants. Among those, 64% (*n* = 315) estimated they had fewer than 10 different contacts, 24% reported between 10 and 19 casual contacts, 6% reported between 20 and 29 contacts and 6% reported an estimated 30 contacts or more.

Individuals who reported high levels (i.e. ≥10 contacts) of casual contacts also tended to report more contacts (Table 1). We found no difference between those reporting high number of casual contacts (≥10) and others, by age, sex or day of the week (Additional file 2: Table S2). However, people whose primary activity was at home tended to report fewer casual contacts than others, and there were about 60% more individuals reporting high levels of casual contacts among those who travelled outside their village.

The 76 (13%) study participants for whom the number of casual contacts was not known known reported more non-casual contacts than others, with a mean number of contacts of 8.7, compared to 7.0 among the 490 for whom the number of casual contacts was estimated (Ratio of means 1.24 (95%CI 1.11 – 1.39). This may be due to the age distribution of individuals for whom estimates of casual contacts was missing, which was proportionally and significantly higher among school aged 5-9 year olds (29% with no information on number of casual contacts), who also report more contacts overall, and lower in all older age groups (Chi-square *P*-value< 0.001).

#### Epidemic simulations

Finally, we compared patterns of reported physical contacts in Uganda and Great Britain, and explored differences in the relative and absolute epidemic size by age group, as well as the corresponding *R*_0_, for a hypothetical respiratory infection in an immune-naive population.

**Fig. 5:**
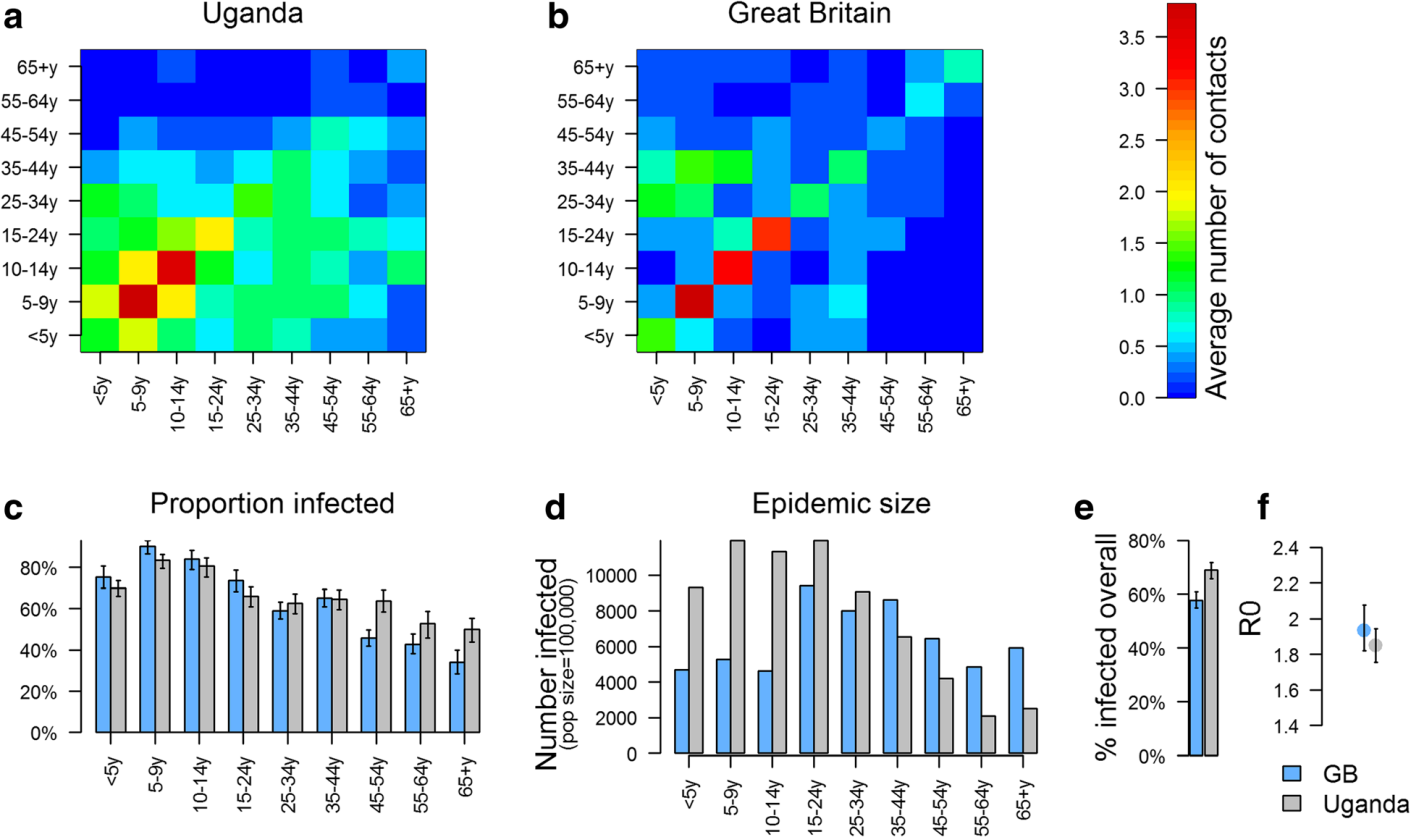
Epidemic Simulation Using Matrices on Physical Contacts from Uganda (**a**) and Great Britain (**b**), for a Hypothetical Respiratory Infection In An Immune-Naïve Population, with the Proportion Infected by Age Group (**c**), the Epidemic Size by Age group (**d**), the Overall Proportion Infected (**d**) and the Basic Reproduction Number R0 (F). **a**: Matrix for physical contacts in Uganda. **b**: Matrix of physical contacts in Great Britain. **c**: Epidemic final size simulation: Proportion of individuals infected by age group in Great Britain (blue) and Uganda (grey), with error bars representing the 95% confidence interval. The results are presented for a *q* value of 33%. **e**: Epidemic size by age group, based on a total population size of 100,000 in Great Britain and in Uganda. **e**: Total proportion of people who were infected at the end of the epidemic in each setting. **f**: Estimates of *R*_0_for each setting, based on a*q* value of 33%, with dots showing the mean value and the bars showing the 95%

The number of reported physical contacts was similar between Uganda and Great Britain, with the average number of contacts by age group ranging from 3.2 (≥65 year olds) to 7.3 (2 – 4 year olds) in Uganda and from 3.3 (55 – 64 year olds) to 7.3 (10 – 14 year olds) in Great Britain. However contacts were more assortative in Britain than in Uganda (Fig. 5a & b), some of which might be related to differences in household structures and number of household contacts, as contacts outside the household were mostly assortative (Fig. 3).

The computed mean values of *R*_0_ for a per contact infectivity value (*q*) ranging from 0.25 to 0.40 was slightly higher in Great Britain than in Uganda (1.51 to 2.41 vs. 1.40 to 2.24). Figure 5f shows the values for an infectivity parameter of 0.33. The proportion of people infected in younger age groups was also higher in Great Britain, and there were proportionally more adults infected in Uganda. However, given the differences in population structure, the total number of infections in the population was higher in Uganda than in Great Britain (Fig. 5c – e).

## Discussion

To our knowledge this is only the fourth study of its kind in Africa [10, 12, 13], and the first one to specifically explore spatial patterns of social contacts. The quantification of mixing patterns is key to accurately model transmission dynamics and inform infectious disease control strategies [4]. Having such data thus fills an important gap, particularly given the high burden of respiratory infections in low income settings [22, 23], and the risk of emerging and re-emerging diseases transmitted by close interpersonal contact, such as influenza [24], measles [25] or meningococcal meningitis [26].

Our findings share similarities with studies from Africa [10, 12, 13] and other low or lower-middle income settings [16, 27], including the high contact frequency among school-aged children and that most contacts tend to be age-assortative. Age-assortativity was not confined to young age groups only, but was also prominent among adults, which contrasts with a recent study from Zimbabwe in which proportional more than age-assortative mixing was reported in older age groups. We also found substantial mixing between age groups, largely driven by intra-household mixing. This may result in a higher force of infection from children to adults than would be seen in high-income settings such as Great Britain, as our final size epidemic model suggests. The final size model should be seen as an illustration of how different social mixing patterns impact on disease epidemiology in different settings, rather than a specific quantification of the differences. It shows the importance of using setting-specific data when modelling disease dynamics and evaluate control strategies. Our data could be best applied to evaluate transmission dynamics and the impact of interventions for endemic diseases and current epidemics in non-naïve populations, for example as for the recent large measles outbreak in the Democratic Republic of the Congo [25]. In our final size model, it is also likely that our retrospective design resulted in underreporting compared to a prospective diary-based approach [28], which hampers comparisons between countries. In sensitivity analyses we explored the impact of potential underreporting in our retrospective survey design compared to a prospective diary-based approach [28], assuming a 25% under-ascertainment compared to a diary-based study, with homogeneous underreporting across age groups. In such scenario, the proportion of infections across all age groups is predicted to be higher in Uganda than in Britain, disproportionally so in adults, and the*R*_0_to be higher too (see Additional file 2: Figure S4).

Our results also provide important insights into the local spatial dynamics of routine daily human interactions in rural Uganda, showing that most contacts tend to occur within the vicinity of people’s area of residence, that working age adult males travel most and young children and the elderly the least, and that contacts tend to be increasingly age assortative as people travel further away from home. Similar patterns were observed in rural and semi-urban China [11]. Such findings have important implications to predict outbreak dynamics and control strategies given that interconnectedness between geographic patches is an essential factor driving epidemic extinction or persistence of epidemics hotspots and the effectiveness of control strategies. Studies of measles in Niger suggest that dynamics differ from that observed in high-income countries in the pre-vaccination era, likely due to different mixing patterns and weaker spatial connectivity [29, 30]. This, together with important variations in vaccination coverage between local geographic patches [31–33], strengthens the need to account for spatial mobility when designing efficient control strategies in those settings. Optimal targeted interventions tailored to specific geographic clusters of high transmission have also been key considerations in recent cholera outbreaks in Africa, given the limited available vaccine doses [34, 35]. Spatially targeted approaches are also central to outbreak control in the recent West African Ebola epidemic [36], and a recent measles epidemic in the Democratic Republic of the Congo, sustained in part due to inadequate coverage of populations in less accessible geographical clusters [25, 37].

Importantly, our data provide a better basis for parameterising transmission models that such spatial dynamics into consideration (meta-population model), by providing quantitative evidence of mixing within and between local areas in a rural East African population.

In our study the frequency of contacts was about half that of the number of contacts reported in Kenya, [10] or South Africa [12]. Although differences between settings are expected, some of these are likely to be due to the exclusion of ‘casual contacts’ from our contact count. There might be further differences linked to the definition of social contacts, which was based on conversational encounters in our study but not in the Kenyan study [10]. When defining contacts based on conversational exchanges the household setting tends to dominate over other settings, compared to a more inclusive definition [8].

Both our contact definition and the retrospective study design may have resulted in more stable, regular contacts being reported over others. However, the extent to which a more inclusive definition reflects contact events relevant for transmission remains unclear. Modelling studies suggest that close interpersonal rather than short casual contacts matter more for transmission of respiratory infections [6]. In addition, for modelling purposes the age-specific structure of relative contact frequency matters more than the actual reported frequency, as matrices are scaled to fit epidemiological data. Our retrospective interview-based design thus offers a simpler and easier alternative to prospective diary based approaches, particularly in such settings. Further research should explore what contact information is most relevant and how such data should best be captured. An additional analysis, which uses data from this contact study with data from the pneumococcal carriage study alongside which our stuy was conducted [15] provides some insight into this question, by exploring contact types associated with pneumococcal carriage and acute respiratory symptoms using data collected at the same time from the same individuals (le Polain de Waroux et al., in preparation and available here [38]).

Selection bias may have occurred to some extent, particularly given that more adult women were included than men. However, there was no significant difference in the number of contacts reported between males and females, including at the weekend, suggesting that selection bias was unlikely to be major. We also tried to reduce selection bias by interviewing on Saturdays people who were initially absent on the survey.

## Conclusion

In conclusion, our study fills an important gap for two main reasons. First, we provide information by detailed age groups about social contacts and mixing patterns relevant to the spread of infectious diseases in a region where such data are scarce. Second, we also provide some insights into spatial characteristics of social encounters. Although this has increasingly being recognized as an important component in evaluating epidemic risk and in the design of efficient control strategies, it has not previously been quantified in low-income settings, and should be explored further. Our study thus provides essential evidence to inform further research and infectious disease modelling work, particularly in similar rural African settings.

## Additional files

Additional file 1: Questionnaire. (DOCX 442 kb)

Additional file 2: Supplementary Tables and Figures. (DOCX 1336 kb)

Additional file 3: Data dictionary. (DOCX 20 kb)

Additional file 4: Data. (CSV 476 kb)

## Abbreviations

BIC: Bayesian Information Criterion
CI: Confidence interval
GPS: Global Positioning Systems
LSHTM: London School of Hygiene and Tropical Medicine
MSF: Médecins Sans Frontières
MUST: Mbarara University of Science and Technology
P: *P*-value
RoM: Ratio of Means
RR: Relative Risk
UNCST: Uganda National Council for Science and Technology

## References

[1] Heesterbeek H, Anderson RM, Andreasen V, Bansal S, De Angelis D, Dye C, Eames KT, Edmunds WJ, Frost SD, Funk S (2015). Modeling infectious disease dynamics in the complex landscape of global health. Science.

[2] Read JM, Edmunds WJ, Riley S, Lessler J, Cummings DA (2012). Close encounters of the infectious kind: methods to measure social mixing behaviour. Epidemiol Infect.

[3] Edmunds WJ, O'Callaghan CJ, Nokes DJ (1997). Who mixes with whom? A method to determine the contact patterns of adults that may lead to the spread of airborne infections. Proc Biol Sci R Soc.

[4] Mossong J, Hens N, Jit M, Beutels P, Auranen K, Mikolajczyk R, Massari M, Salmaso S, Tomba GS, Wallinga J (2008). Social contacts and mixing patterns relevant to the spread of infectious diseases. PLoS Med.

[5] Beutels P, Shkedy Z, Aerts M, Van Damme P (2006). Social mixing patterns for transmission models of close contact infections: exploring self-evaluation and diary-based data collection through a web-based interface. Epidemiol Infect.

[6] Melegaro A, Jit M, Gay N, Zagheni E, Edmunds WJ (2011). What types of contacts are important for the spread of infections?: using contact survey data to explore European mixing patterns. Epidemics.

[7] Smieszek T, Burri EU, Scherzinger R, Scholz RW (2012). Collecting close-contact social mixing data with contact diaries: reporting errors and biases. Epidemiol Infect.

[8] Bolton KJ, McCaw JM, Forbes K, Nathan P, Robins G, Pattison P, Nolan T, McVernon J (2012). Influence of contact definitions in assessment of the relative importance of social settings in disease transmission risk. PLoS One.

[9] Kretzschmar M, Mikolajczyk RT (2009). Contact profiles in eight European countries and implications for modelling the spread of airborne infectious diseases. PLoS One.

[10] Kiti MC, Kinyanjui TM, Koech DC, Munywoki PK, Medley GF, Nokes DJ (2014). Quantifying age-related rates of social contact using diaries in a rural coastal population of Kenya. PLoS One.

[11] Read JM, Lessler J, Riley S, Wang S, Tan LJ, Kwok KO, Guan Y, Jiang CQ, Cummings DA (2014). Social mixing patterns in rural and urban areas of southern China. Proc Biol Sci.

[12] Johnstone-Robertson SP, Mark D, Morrow C, Middelkoop K, Chiswell M, Aquino LD, Bekker LG, Wood R (2011). Social mixing patterns within a south African township community: implications for respiratory disease transmission and control. Am J Epidemiol.

[13] Melegaro A, Del Fava E, Poletti P, Merler S, Nyamukapa C, Williams J, Gregson S, Manfredi P (2017). Social contact structures and time use patterns in the Manicaland Province of Zimbabwe. PLoS One.

[14] Wesolowski A, Metcalf CJ, Eagle N, Kombich J, Grenfell BT, Bjornstad ON, Lessler J, Tatem AJ, Buckee CO (2015). Quantifying seasonal population fluxes driving rubella transmission dynamics using mobile phone data. Proc Natl Acad Sci U S A.

[15] Nackers F, Cohuet S, le Polain de Waroux O, Langendorf C, Nyehangane D, Ndazima D, Nanjebe D, Karani A, Tumwesigye E, Mwanga-Amumpaire J (2017). Carriage prevalence and serotype distribution of Streptococcus pneumoniae prior to 10-valent pneumococcal vaccine introduction: a population-based cross-sectional study in south western Uganda, 2014. Vaccine.

[16] Horby P, Pham QT, Hens N, Nguyen TT, Le QM, Dang DT, Nguyen ML, Nguyen TH, Alexander N, Edmunds WJ (2011). Social contact patterns in Vietnam and implications for the control of infectious diseases. PLoS One.

[17] Rogers WH (1993). Regression standard errors in clustered samples. Stata Tech Bull.

[18] Wallinga J, Teunis P, Kretzschmar M (2006). Using data on social contacts to estimate age-specific transmission parameters for respiratory-spread infectious agents. Am J Epidemiol.

[19] Inglis N, Ross JV, Wilson F, Suleman S, Edeghere O, Smith G, Olowokure B, Keeling MJ, House T (2012). Estimation of outbreak severity and transmissibility: influenza a(H1N1)pdm09 in households. BMC Med.

[20] Kucharski AJ, Kwok KO, Wei VW, Cowling BJ, Read JM, Lessler J, Cummings DA, Riley S (2014). The contribution of social behaviour to the transmission of influenza a in a human population. PLoS Pathog.

[21] The R Project for Statistical Computing. http://www.R-project.org/.

[22] Nair H, Simoes EA, Rudan I, Gessner BD, Azziz-Baumgartner E, Zhang JS, Feikin DR, Mackenzie GA, Moisi JC, Roca A (2013). Global and regional burden of hospital admissions for severe acute lower respiratory infections in young children in 2010: a systematic analysis. Lancet.

[23] Black RE, Cousens S, Johnson HL, Lawn JE, Rudan I, Bassani DG, Jha P, Campbell H, Walker CF, Cibulskis R (2010). Global, regional, and national causes of child mortality in 2008: a systematic analysis. Lancet.

[24] Radin JM, Katz MA, Tempia S, Talla Nzussouo N, Davis R, Duque J, Adedeji A, Adjabeng MJ, Ampofo WK, Ayele W (2012). Influenza surveillance in 15 countries in Africa, 2006-2010. J Infect Dis.

[25] Maurice J (2015). Measles outbreak in DR Congo an “epidemic emergency”. Lancet.

[26] Burki T (2015). Meningitis outbreak in Niger is an urgent warning. Lancet Infect Dis.

[27] Grijalva CG, Goeyvaerts N, Verastegui H, Edwards KM, Gil AI, Lanata CF, Hens N, Project RP (2015). A household-based study of contact networks relevant for the spread of infectious diseases in the highlands of Peru. PLoS One.

[28] McCaw JM, Forbes K, Nathan PM, Pattison PE, Robins GL, Nolan TM, McVernon J (2010). Comparison of three methods for ascertainment of contact information relevant to respiratory pathogen transmission in encounter networks. BMC Infect Dis.

[29] Grais RF, Ferrari MJ, Dubray C, Bjornstad ON, Grenfell BT, Djibo A, Fermon F, Guerin PJ (2006). Estimating transmission intensity for a measles epidemic in Niamey, Niger: lessons for intervention. Trans R Soc Trop Med Hyg.

[30] Ferrari MJ, Grais RF, Bharti N, Conlan AJ, Bjornstad ON, Wolfson LJ, Guerin PJ, Djibo A, Grenfell BT (2008). The dynamics of measles in sub-Saharan Africa. Nature.

[31] Le Polain de Waroux O, Schellenberg JR, Manzi F, Mrisho M, Shirima K, Mshinda H, Alonso P, Tanner M, Schellenberg DM (2013). Timeliness and completeness of vaccination and risk factors for low and late vaccine uptake in young children living in rural southern Tanzania. Int Health.

[32] Babirye JN, Engebretsen IM, Makumbi F, Fadnes LT, Wamani H, Tylleskar T, Nuwaha F (2012). Timeliness of childhood vaccinations in Kampala Uganda: a community-based cross-sectional study. PLoS One.

[33] Satzke C, Turner P, Virolainen-Julkunen A, Adrian PV, Antonio M, Hare KM, Henao-Restrepo AM, Leach AJ, Klugman KP, Porter BD (2013). Standard method for detecting upper respiratory carriage of Streptococcus pneumoniae: updated recommendations from the World Health Organization pneumococcal carriage working group. Vaccine.

[34] Azman AS, Luquero FJ, Rodrigues A, Palma PP, Grais RF, Banga CN, Grenfell BT, Lessler J (2012). Urban cholera transmission hotspots and their implications for reactive vaccination: evidence from Bissau city, Guinea Bissau. PLoS Negl Trop Dis.

[35] Luquero FJ, Banga CN, Remartinez D, Palma PP, Baron E, Grais RF (2011). Cholera epidemic in Guinea-Bissau (2008): the importance of “place”. PLoS One.

[36] Zinszer K, Morrison K, Anema A, Majumder MS, Brownstein JS (2015). The velocity of Ebola spread in parts of West Africa. Lancet Infect Dis.

[37] Gil Cuesta J, Mukembe N, Valentiner-Branth P, Stefanoff P, Lenglet A: Measles vaccination coverage survey in moba, katanga, democratic republic of congo, 2013: need to adapt routine and mass vaccination campaigns to reach the unreached. PLoS Curr. 2015;7. 10.1371/currents.outbreaks.8a1b00760dfd81481eb42234bd18ced3. 10.1371/currents.outbreaks.8a1b00760dfd81481eb42234bd18ced3 PMC4336195 25713744

[38] Le Polain de Waroux O, Flasche S, Kucharksi AJ, Langendorf C, Ndazima D, Mwanga-Amumpaire J, Grais RF, Cohuet S, Edmunds WJ (2017). Identifying human encounters that shape the transmission of Streptococcus pneumoniae and other respiratory infections.

