# Supplementary material for: Characteristics of human encounters and social mixing patterns relevant to infectious diseases spread by close contact: a survey in Southwest Uganda
Source: BMC Infect Dis. 2018 Apr 11;18:172. doi: 10.1186/s12879-018-3073-1 (PMC5896105; doi:10.1186/s12879-018-3073-1)
Supplement: Supplementary file 1 — Questionnaire. (DOCX 442 kb) [file 12879_2018_3073_MOESM1_ESM.docx]

### Additional File 1: Social contact questionnaire

There are three parts to this questionnaire. We will first ask a few questions about yourself/your child and the people you live with. Second, we will ask you questions about the different places you/your child went to yesterday. Finally, we will ask you to remember who you/your child met during the day in each of the places you attended, and how long for.

1. *[ID and dates]*
   1. *[Individual participant ID]:* **|**__|__**|**__|__**|**
   2. *[Interview date (dd/mm/yy) ]*: **|**__|__**|**__|__**|**__|__**|**
   3. *[Date of the “surveyed day” (dd/mm/yy)]* : **|**__|__**|**__|__**|**__|__**|**
   4. *[Day of the week of the “surveyed day”]*

1= Monday

2= Tuesday

3= Wednesday

4= Thursday

5= Friday

6= Saturday

7= Sunday

**PART ONE: SOCIO-DEMOGRAPHIC CHARACTERISTICS**

1. We would first like to ask a few questions about yourself/your child as well as about the people living with you/your child in your household.
   1. Age **|**__|__**|** years
   2. *[Sex of the participant ]*

1= Male

2 = Female

- 1. What is your/your child’s primary occupation or daily activity *[note to the interviewer: this is the activity the participant spends the most time doing on a daily basis]?*

1 = pre-school child;

2= school/college/university student

3= office worker

4= shop worker

5= agriculture

6= manual worker

7= housewife

8= unemployed

9= retired

10= others

-1= don’t know

-2 = refused

- 1. Including yourself/your child, how many people live in your household? **A household is the group of individuals living under the same roof and sharing the same kitchen on a daily basis**

**|**__|__**|**. *[-1 for don’t know and -2 for refused]*

- 1. How many bedrooms are in your household (only count living and sleeping rooms, exclude bathroom, kitchen)? **|**__|__**|** *[-1 for don’t know and -2 for refused]*

1. We will now ask three questions about contacts you may have with animals
   1. Do you/your child touch the following animals at least once per week?:

1= chickens, ducks, gees

2= cows, pigs, goats, sheep

3= rodent

4= primates

5= bats

6= antelope

7= none of the above

- 1. In the last month, have you/has your child been bitten or scratched or cut by any of these animals?

1= chickens, ducks, gees

2= cows, pigs, goats, sheep

3= rodent

4= primates

5= bats

6= antelope

7= none of the above

- 1. In the last month, have you/has your child killed, butchered or cooked any of these animals?

1= chickens, ducks, geese

2= cows, pigs, goats, sheep

3= none of the above

1. Including yourself/your child, could you please list all individuals in your household, as well as the family links between yourself/your child and each of the household members. ***[define the household again]***

| **Household member initials/name** | **Household member ID** | | | **Sex** | | **Age in years** | **Relationship** | | | | | | | | |
| --- | --- | --- | --- | --- | --- | --- | --- | --- | --- | --- | --- | --- | --- | --- | --- |
|  |  |  |  |  |  |  | me | Spouse | Sibling | Child | Parent | Grandparent | Uncle/Aunt | Other family | Unrelated |
|  |  |  |  | **M=1** | **F=2** |  |  |  |  |  |  |  |  |  |  |
|  | H | 0 | 1 | 1 | 2 |  | 1 | 2 | 3 | 4 | 5 | 6 | 7 | 8 | 9 |
|  | H |  |  | 1 | 2 |  | 1 | 2 | 3 | 4 | 5 | 6 | 7 | 8 | 9 |
|  | H |  |  | 1 | 2 |  | 1 | 2 | 3 | 4 | 5 | 6 | 7 | 8 | 9 |
|  | H |  |  | 1 | 2 |  | 1 | 2 | 3 | 4 | 5 | 6 | 7 | 8 | 9 |
|  | H |  |  | 1 | 2 |  | 1 | 2 | 3 | 4 | 5 | 6 | 7 | 8 | 9 |
|  | H |  |  | 1 | 2 |  | 1 | 2 | 3 | 4 | 5 | 6 | 7 | 8 | 9 |
|  | H |  |  | 1 | 2 |  | 1 | 2 | 3 | 4 | 5 | 6 | 7 | 8 | 9 |
|  | H |  |  | 1 | 2 |  | 1 | 2 | 3 | 4 | 5 | 6 | 7 | 8 | 9 |
|  | H |  |  | 1 | 2 |  | 1 | 2 | 3 | 4 | 5 | 6 | 7 | 8 | 9 |
|  | H |  |  | 1 | 2 |  | 1 | 2 | 3 | 4 | 5 | 6 | 7 | 8 | 9 |
|  | H |  |  | 1 | 2 |  | 1 | 2 | 3 | 4 | 5 | 6 | 7 | 8 | 9 |
|  | H |  |  | 1 | 2 |  | 1 | 2 | 3 | 4 | 5 | 6 | 7 | 8 | 9 |
|  | H |  |  | 1 | 2 |  | 1 | 2 | 3 | 4 | 5 | 6 | 7 | 8 | 9 |
|  | H |  |  | 1 | 2 |  | 1 | 2 | 3 | 4 | 5 | 6 | 7 | 8 | 9 |
|  | H |  |  | 1 | 2 |  | 1 | 2 | 3 | 4 | 5 | 6 | 7 | 8 | 9 |
|  | H |  |  | 1 | 2 |  | 1 | 2 | 3 | 4 | 5 | 6 | 7 | 8 | 9 |
|  | H |  |  | 1 | 2 |  | 1 | 2 | 3 | 4 | 5 | 6 | 7 | 8 | 9 |

**PART TWO: SETTINGS**

1. How far do you/your child travel(s) outside your village or town and how often?

| **Geographical setting** | **How often to you travel to those places outside your village or town?** | | | | | | **How long do you spend in that place when you go** | | | | |
| --- | --- | --- | --- | --- | --- | --- | --- | --- | --- | --- | --- |
|  | Most days of the weeks | At least once a week | At least once a month, but not each week | Less than once a month | < once a month | Never | <1h | 1-2h | Half a day | A whole day | Never |
| To a place in another village/town <5km away (<1 hour walk) | 1 | 2 | 3 | 4 | 5 | 6 | 1 | 2 | 3 | 4 | 5 |
| To a place in another village/town ≥5km away | 1 | 2 | 3 | 4 | 5 | 6 | 1 | 2 | 3 | 4 | 5 |

1. In the last week,
   1. What is the name of the village/town of the furthest place from home you/your child went to? ______________________________
   2. [Write the geographic ID of the abovementioned village/town **(see geographic ID form)**] **|**__|__**|**
2. Where did you/ your spend child time yesterday **[between the time of wake up yesterday and the time of wake up today]**

| **Setting ID** | | | **Type of place** | | | | | | | | | | If Other, where did you go? *[free text]* | **Name of the village or town** |  | | | **Time spent in that place** | | | | |
| --- | --- | --- | --- | --- | --- | --- | --- | --- | --- | --- | --- | --- | --- | --- | --- | --- | --- | --- | --- | --- | --- | --- |
|  |  |  | Home | Another house | work | School | Place of worship | Transport | Leisure | Shop | Garden | Other |  |  | **Village/town with geographic ID** | | | <15mins | 15mins – <1h | 1 - <2h | 2-<4h | >4h |
| S | 0 | 1 | 1 | 2 | 3 | 4 | 5 | 6 | 7 | 8 | 9 | 10 |  |  |  |  |  | 1 | 2 | 3 | 4 | 5 |
| S | 0 | 2 | 1 | 2 | 3 | 4 | 5 | 6 | 7 | 8 | 9 | 10 |  |  |  |  |  | 1 | 2 | 3 | 4 | 5 |
| S | 0 | 3 | 1 | 2 | 3 | 4 | 5 | 6 | 7 | 8 | 9 | 10 |  |  |  |  |  | 1 | 2 | 3 | 4 | 5 |
| S | 0 | 4 | 1 | 2 | 3 | 4 | 5 | 6 | 7 | 8 | 9 | 10 |  |  |  |  |  | 1 | 2 | 3 | 4 | 5 |
| S | 0 | 5 | 1 | 2 | 3 | 4 | 5 | 6 | 7 | 8 | 9 | 10 |  |  |  |  |  | 1 | 2 | 3 | 4 | 5 |
| S | 0 | 6 | 1 | 2 | 3 | 4 | 5 | 6 | 7 | 8 | 9 | 10 |  |  |  |  |  | 1 | 2 | 3 | 4 | 5 |
| S | 0 | 7 | 1 | 2 | 3 | 4 | 5 | 6 | 7 | 8 | 9 | 10 |  |  |  |  |  | 1 | 2 | 3 | 4 | 5 |
| S | 0 | 8 | 1 | 2 | 3 | 4 | 5 | 6 | 7 | 8 | 9 | 10 |  |  |  |  |  | 1 | 2 | 3 | 4 | 5 |
| S | 0 | 9 | 1 | 2 | 3 | 4 | 5 | 6 | 7 | 8 | 9 | 10 |  |  |  |  |  | 1 | 2 | 3 | 4 | 5 |
| S | 1 | 0 | 1 | 2 | 3 | 4 | 5 | 6 | 7 | 8 | 9 | 10 |  |  |  |  |  | 1 | 2 | 3 | 4 | 5 |
| S | 1 | 1 | 1 | 2 | 3 | 4 | 5 | 6 | 7 | 8 | 9 | 10 |  |  |  |  |  | 1 | 2 | 3 | 4 | 5 |
| S | 1 | 2 | 1 | 2 | 3 | 4 | 5 | 6 | 7 | 8 | 9 | 10 |  |  |  |  |  | 1 | 2 | 3 | 4 | 5 |
| S | 1 | 3 | 1 | 2 | 3 | 4 | 5 | 6 | 7 | 8 | 9 | 10 |  |  |  |  |  | 1 | 2 | 3 | 4 | 5 |
| S | 1 | 4 | 1 | 2 | 3 | 4 | 5 | 6 | 7 | 8 | 9 | 10 |  |  |  |  |  | 1 | 2 | 3 | 4 | 5 |
| S | 1 | 5 | 1 | 2 | 3 | 4 | 5 | 6 | 7 | 8 | 9 | 10 |  |  |  |  |  | 1 | 2 | 3 | 4 | 5 |
| S | 1 | 6 | 1 | 2 | 3 | 4 | 5 | 6 | 7 | 8 | 9 | 10 |  |  |  |  |  | 1 | 2 | 3 | 4 | 5 |
| S | 2 | 0 | 1 | 2 | 3 | 4 | 5 | 6 | 7 | 8 | 9 | 10 |  |  |  |  |  | 1 | 2 | 3 | 4 | 5 |

**PART THREE: CONTACTS**

We will now ask you to remember who you were/your child was in contact with yesterday **[between the time of wake up yesterday and the time of wake up today].**

1. How many people have you/your child seen **for a very short period of time (<5mins)** and with whom you/your child have **exchanged at least three words** yesterday? (e.g. saying hello to someone on your way, seeing someone in a shop, seeing a few children at school in the playground, etc)

1= 0 – 9 people

2= 10 – 19 people

3= 20 – 29 people

4= >30 people

5= don’t know

1. **Contacts**

We would now like you to remember any person with whom you spent 5 minutes or more and with whom you exchanged at least three words in each of those settings. Those individuals will be defined as your contacts. For each of them, we would like to know if you had physical contact or non-physical contact. Nonphysical contact happens when you haven’t touched the person. Physical contact includes hand shaking, sharing a bike, kissing, embracing, and also sharing a glass or other utensils passed directly from mouth to mouth.

9.1 We will first ask a few more questions about the people you were/your child was in contact with **at home** yesterday **for more than 5 minutes** ***[yesterday is defined as the period from wake up yesterdat to the moment you woke up this morning]***

| **Contact initials/name**  **[will be removed from the questionnaire after the interview]** | **Contact ID** | | | **Place ID** | | | Age (yrs) | **Sex (M/F)** | | **Type of contact** | | **Link to the contact** | | | | | **How often do you have contact with this person in general?** | | | | | **Total time spent with the person in that particular place** | | | | |
| --- | --- | --- | --- | --- | --- | --- | --- | --- | --- | --- | --- | --- | --- | --- | --- | --- | --- | --- | --- | --- | --- | --- | --- | --- | --- | --- |
|  |  |  |  |  |  |  |  | M=1 | F=2 | Physical | Nonphysical | Household member | Other relative | colleague or schoolmate | Friend | Other | Daily or almost daily | At least once a week | At least once a month | Less than once a month | Never met before | 5 - <15 mins | 15 – <1h | 1h - <2h | 2h - <4h | >4h |
|  | C | 0 | 1 | S | 0 | 1 |  | 1 | 2 | 1 | 2 | 1 | 2 | 3 | 4 | 5 | 1 | 2 | 3 | 4 | 5 | 1 | 2 | 3 | 4 | 5 |
|  | C |  |  | S | 0 | 1 |  | 1 | 2 | 1 | 2 | 1 | 2 | 3 | 4 | 5 | 1 | 2 | 3 | 4 | 5 | 1 | 2 | 3 | 4 | 5 |
|  | C |  |  | S | 0 | 1 |  | 1 | 2 | 1 | 2 | 1 | 2 | 3 | 4 | 5 | 1 | 2 | 3 | 4 | 5 | 1 | 2 | 3 | 4 | 5 |
|  | C |  |  | S | 0 | 1 |  | 1 | 2 | 1 | 2 | 1 | 2 | 3 | 4 | 5 | 1 | 2 | 3 | 4 | 5 | 1 | 2 | 3 | 4 | 5 |
|  | C |  |  | S | 0 | 1 |  | 1 | 2 | 1 | 2 | 1 | 2 | 3 | 4 | 5 | 1 | 2 | 3 | 4 | 5 | 1 | 2 | 3 | 4 | 5 |
|  | C |  |  | S | 0 | 1 |  | 1 | 2 | 1 | 2 | 1 | 2 | 3 | 4 | 5 | 1 | 2 | 3 | 4 | 5 | 1 | 2 | 3 | 4 | 5 |
|  | C |  |  | S | 0 | 1 |  | 1 | 2 | 1 | 2 | 1 | 2 | 3 | 4 | 5 | 1 | 2 | 3 | 4 | 5 | 1 | 2 | 3 | 4 | 5 |
|  | C |  |  | S | 0 | 1 |  | 1 | 2 | 1 | 2 | 1 | 2 | 3 | 4 | 5 | 1 | 2 | 3 | 4 | 5 | 1 | 2 | 3 | 4 | 5 |
|  | C |  |  | S | 0 | 1 |  | 1 | 2 | 1 | 2 | 1 | 2 | 3 | 4 | 5 | 1 | 2 | 3 | 4 | 5 | 1 | 2 | 3 | 4 | 5 |
|  | C |  |  | S | 0 | 1 |  | 1 | 2 | 1 | 2 | 1 | 2 | 3 | 4 | 5 | 1 | 2 | 3 | 4 | 5 | 1 | 2 | 3 | 4 | 5 |
|  | C |  |  | S | 0 | 1 |  | 1 | 2 | 1 | 2 | 1 | 2 | 3 | 4 | 5 | 1 | 2 | 3 | 4 | 5 | 1 | 2 | 3 | 4 | 5 |
|  | C |  |  | S | 0 | 1 |  | 1 | 2 | 1 | 2 | 1 | 2 | 3 | 4 | 5 | 1 | 2 | 3 | 4 | 5 | 1 | 2 | 3 | 4 | 5 |
|  | C |  |  | S | 0 | 1 |  | 1 | 2 | 1 | 2 | 1 | 2 | 3 | 4 | 5 | 1 | 2 | 3 | 4 | 5 | 1 | 2 | 3 | 4 | 5 |
|  | C |  |  | S | 0 | 1 |  | 1 | 2 | 1 | 2 | 1 | 2 | 3 | 4 | 5 | 1 | 2 | 3 | 4 | 5 | 1 | 2 | 3 | 4 | 5 |
|  | C |  |  | S | 0 | 1 |  | 1 | 2 | 1 | 2 | 1 | 2 | 3 | 4 | 5 | 1 | 2 | 3 | 4 | 5 | 1 | 2 | 3 | 4 | 5 |
|  | C |  |  | S | 0 | 1 |  | 1 | 2 | 1 | 2 | 1 | 2 | 3 | 4 | 5 | 1 | 2 | 3 | 4 | 5 | 1 | 2 | 3 | 4 | 5 |
|  | C |  |  | S | 0 | 1 |  | 1 | 2 | 1 | 2 | 1 | 2 | 3 | 4 | 5 | 1 | 2 | 3 | 4 | 5 | 1 | 2 | 3 | 4 | 5 |
|  | C |  |  | S | 0 | 1 |  | 1 | 2 | 1 | 2 | 1 | 2 | 3 | 4 | 5 | 1 | 2 | 3 | 4 | 5 | 1 | 2 | 3 | 4 | 5 |
|  | C |  |  | S | 0 | 1 |  | 1 | 2 | 1 | 2 | 1 | 2 | 3 | 4 | 5 | 1 | 2 | 3 | 4 | 5 | 1 | 2 | 3 | 4 | 5 |
|  |  |  |  |  |  |  |  |  |  |  |  |  |  |  |  |  |  |  |  |  |  |  |  |  |  |  |

- 1. We will now ask a few more questions about the people you were/your child was in contact with in the other places yesterday for more than 5 minutes

| **Contact initials/name** | **Contact number ID** | | | **Place ID** | | | Age (yrs) | **Sex (M/F)** | | **Type of contact** | | **Link to the contact** | | | | | **How often do you have contact with this person in general?** | | | | | **Total time spent with the person in that particular place** | | | | |
| --- | --- | --- | --- | --- | --- | --- | --- | --- | --- | --- | --- | --- | --- | --- | --- | --- | --- | --- | --- | --- | --- | --- | --- | --- | --- | --- |
|  |  |  |  |  |  |  |  | M=1 | F=2 | Close - physical | Close - nonphsycial | Household member | Other relative | colleague or schoolmate | Friend | Other | Daily or almost daily | At least once a week | At least once a month | Less than once a month | Never met before | 5 - <15 mins | 15 – <1h | 1h - <2h | 2h - <4h | >4h |
|  | C |  |  | S |  |  |  | 1 | 2 | 1 | 2 | 1 | 2 | 3 | 4 | 5 | 1 | 2 | 3 | 4 | 5 | 1 | 2 | 3 | 4 | 5 |
|  | C |  |  | S |  |  |  | 1 | 2 | 1 | 2 | 1 | 2 | 3 | 4 | 5 | 1 | 2 | 3 | 4 | 5 | 1 | 2 | 3 | 4 | 5 |
|  | C |  |  | S |  |  |  | 1 | 2 | 1 | 2 | 1 | 2 | 3 | 4 | 5 | 1 | 2 | 3 | 4 | 5 | 1 | 2 | 3 | 4 | 5 |
|  | C |  |  | S |  |  |  | 1 | 2 | 1 | 2 | 1 | 2 | 3 | 4 | 5 | 1 | 2 | 3 | 4 | 5 | 1 | 2 | 3 | 4 | 5 |
|  | C |  |  | S |  |  |  | 1 | 2 | 1 | 2 | 1 | 2 | 3 | 4 | 5 | 1 | 2 | 3 | 4 | 5 | 1 | 2 | 3 | 4 | 5 |
|  | C |  |  | S |  |  |  | 1 | 2 | 1 | 2 | 1 | 2 | 3 | 4 | 5 | 1 | 2 | 3 | 4 | 5 | 1 | 2 | 3 | 4 | 5 |
|  | C |  |  | S |  |  |  | 1 | 2 | 1 | 2 | 1 | 2 | 3 | 4 | 5 | 1 | 2 | 3 | 4 | 5 | 1 | 2 | 3 | 4 | 5 |
|  | C |  |  | S |  |  |  | 1 | 2 | 1 | 2 | 1 | 2 | 3 | 4 | 5 | 1 | 2 | 3 | 4 | 5 | 1 | 2 | 3 | 4 | 5 |
|  | C |  |  | S |  |  |  | 1 | 2 | 1 | 2 | 1 | 2 | 3 | 4 | 5 | 1 | 2 | 3 | 4 | 5 | 1 | 2 | 3 | 4 | 5 |
|  | C |  |  | S |  |  |  | 1 | 2 | 1 | 2 | 1 | 2 | 3 | 4 | 5 | 1 | 2 | 3 | 4 | 5 | 1 | 2 | 3 | 4 | 5 |
|  | C |  |  | S |  |  |  | 1 | 2 | 1 | 2 | 1 | 2 | 3 | 4 | 5 | 1 | 2 | 3 | 4 | 5 | 1 | 2 | 3 | 4 | 5 |
|  | C |  |  | S |  |  |  | 1 | 2 | 1 | 2 | 1 | 2 | 3 | 4 | 5 | 1 | 2 | 3 | 4 | 5 | 1 | 2 | 3 | 4 | 5 |
|  | C |  |  | S |  |  |  | 1 | 2 | 1 | 2 | 1 | 2 | 3 | 4 | 5 | 1 | 2 | 3 | 4 | 5 | 1 | 2 | 3 | 4 | 5 |
|  | C |  |  | S |  |  |  | 1 | 2 | 1 | 2 | 1 | 2 | 3 | 4 | 5 | 1 | 2 | 3 | 4 | 5 | 1 | 2 | 3 | 4 | 5 |
|  | C |  |  | S |  |  |  | 1 | 2 | 1 | 2 | 1 | 2 | 3 | 4 | 5 | 1 | 2 | 3 | 4 | 5 | 1 | 2 | 3 | 4 | 5 |
|  | C |  |  | S |  |  |  | 1 | 2 | 1 | 2 | 1 | 2 | 3 | 4 | 5 | 1 | 2 | 3 | 4 | 5 | 1 | 2 | 3 | 4 | 5 |
|  | C |  |  | S |  |  |  | 1 | 2 | 1 | 2 | 1 | 2 | 3 | 4 | 5 | 1 | 2 | 3 | 4 | 5 | 1 | 2 | 3 | 4 | 5 |
|  | C |  |  | S |  |  |  | 1 | 2 | 1 | 2 | 1 | 2 | 3 | 4 | 5 | 1 | 2 | 3 | 4 | 5 | 1 | 2 | 3 | 4 | 5 |
|  | C |  |  | S |  |  |  | 1 | 2 | 1 | 2 | 1 | 2 | 3 | 4 | 5 | 1 | 2 | 3 | 4 | 5 | 1 | 2 | 3 | 4 | 5 |
|  | C |  |  | S |  |  |  | 1 | 2 | 1 | 2 | 1 | 2 | 3 | 4 | 5 | 1 | 2 | 3 | 4 | 5 | 1 | 2 | 3 | 4 | 5 |
|  | C |  |  | S |  |  |  | 1 | 2 | 1 | 2 | 1 | 2 | 3 | 4 | 5 | 1 | 2 | 3 | 4 | 5 | 1 | 2 | 3 | 4 | 5 |
|  | C |  |  | S |  |  |  | 1 | 2 | 1 | 2 | 1 | 2 | 3 | 4 | 5 | 1 | 2 | 3 | 4 | 5 | 1 | 2 | 3 | 4 | 5 |
|  | C |  |  | S |  |  |  | 1 | 2 | 1 | 2 | 1 | 2 | 3 | 4 | 5 | 1 | 2 | 3 | 4 | 5 | 1 | 2 | 3 | 4 | 5 |
|  | C |  |  | S |  |  |  | 1 | 2 | 1 | 2 | 1 | 2 | 3 | 4 | 5 | 1 | 2 | 3 | 4 | 5 | 1 | 2 | 3 | 4 | 5 |
|  | C |  |  | S |  |  |  | 1 | 2 | 1 | 2 | 1 | 2 | 3 | 4 | 5 | 1 | 2 | 3 | 4 | 5 | 1 | 2 | 3 | 4 | 5 |
|  | C |  |  | S |  |  |  | 1 | 2 | 1 | 2 | 1 | 2 | 3 | 4 | 5 | 1 | 2 | 3 | 4 | 5 | 1 | 2 | 3 | 4 | 5 |
|  | C |  |  | S |  |  |  | 1 | 2 | 1 | 2 | 1 | 2 | 3 | 4 | 5 | 1 | 2 | 3 | 4 | 5 | 1 | 2 | 3 | 4 | 5 |
|  | C |  |  | S |  |  |  | 1 | 2 | 1 | 2 | 1 | 2 | 3 | 4 | 5 | 1 | 2 | 3 | 4 | 5 | 1 | 2 | 3 | 4 | 5 |
|  | C |  |  | S |  |  |  | 1 | 2 | 1 | 2 | 1 | 2 | 3 | 4 | 5 | 1 | 2 | 3 | 4 | 5 | 1 | 2 | 3 | 4 | 5 |
|  | C |  |  | S |  |  |  | 1 | 2 | 1 | 2 | 1 | 2 | 3 | 4 | 5 | 1 | 2 | 3 | 4 | 5 | 1 | 2 | 3 | 4 | 5 |
|  | C |  |  | S |  |  |  | 1 | 2 | 1 | 2 | 1 | 2 | 3 | 4 | 5 | 1 | 2 | 3 | 4 | 5 | 1 | 2 | 3 | 4 | 5 |
|  | C |  |  | S |  |  |  | 1 | 2 | 1 | 2 | 1 | 2 | 3 | 4 | 5 | 1 | 2 | 3 | 4 | 5 | 1 | 2 | 3 | 4 | 5 |
|  | C |  |  | S |  |  |  | 1 | 2 | 1 | 2 | 1 | 2 | 3 | 4 | 5 | 1 | 2 | 3 | 4 | 5 | 1 | 2 | 3 | 4 | 5 |
|  | C |  |  | S |  |  |  | 1 | 2 | 1 | 2 | 1 | 2 | 3 | 4 | 5 | 1 | 2 | 3 | 4 | 5 | 1 | 2 | 3 | 4 | 5 |
|  | C |  |  | S |  |  |  | 1 | 2 | 1 | 2 | 1 | 2 | 3 | 4 | 5 | 1 | 2 | 3 | 4 | 5 | 1 | 2 | 3 | 4 | 5 |
|  | C |  |  | S |  |  |  | 1 | 2 | 1 | 2 | 1 | 2 | 3 | 4 | 5 | 1 | 2 | 3 | 4 | 5 | 1 | 2 | 3 | 4 | 5 |
|  | C |  |  | S |  |  |  | 1 | 2 | 1 | 2 | 1 | 2 | 3 | 4 | 5 | 1 | 2 | 3 | 4 | 5 | 1 | 2 | 3 | 4 | 5 |
|  | C |  |  | S |  |  |  | 1 | 2 | 1 | 2 | 1 | 2 | 3 | 4 | 5 | 1 | 2 | 3 | 4 | 5 | 1 | 2 | 3 | 4 | 5 |
|  | C |  |  | S |  |  |  | 1 | 2 | 1 | 2 | 1 | 2 | 3 | 4 | 5 | 1 | 2 | 3 | 4 | 5 | 1 | 2 | 3 | 4 | 5 |
|  | C |  |  | S |  |  |  | 1 | 2 | 1 | 2 | 1 | 2 | 3 | 4 | 5 | 1 | 2 | 3 | 4 | 5 | 1 | 2 | 3 | 4 | 5 |
|  | C |  |  | S |  |  |  | 1 | 2 | 1 | 2 | 1 | 2 | 3 | 4 | 5 | 1 | 2 | 3 | 4 | 5 | 1 | 2 | 3 | 4 | 5 |
|  | C |  |  | S |  |  |  | 1 | 2 | 1 | 2 | 1 | 2 | 3 | 4 | 5 | 1 | 2 | 3 | 4 | 5 | 1 | 2 | 3 | 4 | 5 |
|  | C |  |  | S |  |  |  | 1 | 2 | 1 | 2 | 1 | 2 | 3 | 4 | 5 | 1 | 2 | 3 | 4 | 5 | 1 | 2 | 3 | 4 | 5 |

Note that for the survey there was no limit to the number of contacts that could be entered as surveyors were able to add additional sheets as needed.

A slightly adapted questionnaire was used for children and parents.

### Supporting File 2: Guidelines for the survey team

*Note that for clarity here is a summary of the guidelines for the social contact survey part of the entire survey (which included both a nasopharyngeal carriage survey, with nasopharyngeal sampling procedures, collection and transport of specimens etc.)*

The field coordinator will call the Village Health Teams to ensure their help for data collection during the day of the survey (which will take place 48h later) in the village. VHTs will be told that they will receive incentives to participate in the survey. They will be in charge to inform the heads of the selected households in the village/ cluster that the survey teams will visit their household two days later and that they should try to remember their social encounters during the 24 hours preceding the survey day. For households for which a telephone number is available, the head of household will be also directly contacted by the study coordinator to notify them of the survey team visit two days later.

The teamss will be divided in two groups of three and two teams (A + B + C and D+E) . The teams will go by motorbike to visit the selected villages of the day. Teams will visit 10 households per day in one same village, and D and E will visit 15 households per day. Fewer households need to be visited by teams A, B and C as they will visit villages located further away and/or more sparsely populated whereas teams D and E will visit villages that are easier to access and survey.

The list of households to be visited by village/cluster, including names of the head of households, will be available. **A household is defined as people who are sleeping under the same roof and sharing the same meal on a daily basis**. No matter the age of the household members or the relation between the members.

**Selection of households**

The nurse and the VHT will introduce the team to the household head or to another adult member of the family (i.e. anyone aged 18 years or older) in their absence. If either no adult or nobody is present in the household at the time of the visit, the team will move to the next household on their list and will come back to the earlier household later during the day.

If the head of the household or the participant refuses to participate in the survey, or if swabbing is contra-indicated, the household will be replaced by another household from a replacement list provided (**see list of households form**).

At 6pm the activities in the field will stop. Teams will then assess the number of households that were not available to visit and feed this information back to the field coordinator . The field coordinator will then call the household heads of the households that were missed to inform them again about the survey and tell them that teams will come to visit them again on the Saturday of the same week. If the head of the household tells the field coordinator that no one will be present on the Saturday, or if the head of the household cannot be reached by telephone, those households will be replaced from a random list(**see list of households form**) , and contact will be made by the field coordinator with those replaced households.

The household head will be carefully introduced to the survey purpose. Social benefit and confidentiality should be strongly emphasized (see **information participant form**). The verbal consent of the head of the household should be obtained. If the head of the household refuses to participate, teams should not insist on their participation. Teams should make a note about their refusal on the list of households (refusal yes/no) and continue with the next household on the list.

**Selection of participants within households**

- All individuals who are members of the selected households in the village/cluster will be eligible for study participation except if:

1. the participant or their representative either refuses or is not able to provide informed consent to participate
2. if they have head or facial injuries that would contraindicate NP swabbing.

- Only one person will be selected per household. To ensure that the sample size is reached for each age group, the age group of the individual to select will be predetermined using the “age group selection for cluster form” (see **age group selection for cluster form**). If several individuals of the same required age group are members of the selected household, the person to be included in the survey will be randomly chosen.
- If no one in the household is from the selected age group, the surveyors are entitled to include someone from another age group instead, so long as the sampling quota has not been reached for all other age groups from the list of households to include.
- If no one can be included, the household should be replaced by another household from the replacement list (**see list of households form**).
- Once the individual is selected within the household, the individual or the caregiver of a person less than 18 years old will be carefully introduced to the survey purpose by the nurse **(**see **information participant form).**
- Written consent from the individual has to be obtained (see **consent form over18 and consent formunder18**). If the selected individual refuses to participate, do not insist on their participation. If another household member in the same age group agrees participation, undertake the survey with them. Otherwise, move to the next household on the list.
- When the selected member of the household or their caregiver has given written consent to participate in the survey the interviewer will take the GPS coordinates of the household (see **SOP GPS coordinates**).

# **Contact survey questionnaire**

1. **General comments**

- You (the interviewer) will have to complete the contact survey questionnaire for the individuals marked with a cross in the **age group selection for cluster form.**
- There are three versions of the questionnaire, each of which are adapted to particular age groups: <5y olds, 5-14y olds and ≥15y olds.
- Each team will carry one example of each questionnaire in Ruyankole. The interview will be carried out in Ruyankole, but questionnaires should be completed in English. The interviewer will conduct the interviews, and the study nurse will record the information on the English questionnaire.
- For children <5 years of age, parents/guardians will be interviewed on their behalf. Use the appropriate questionnaire for this. When asking questions about their child, replace ‘your child’ with the child’s name
- Any information for the interviewer or any question that can be filled in directly be the interviewer without the help of the interviewee (e.g. date, sex of the interviewee) are shown in italics between squared brackets on the questionnaire
- For questions with multiple choices, please circle the appropriate response. In case of mistake, strike through the answer and circle the correct one, with a little ‘V’ sign next to it to notify the change, as shown here below


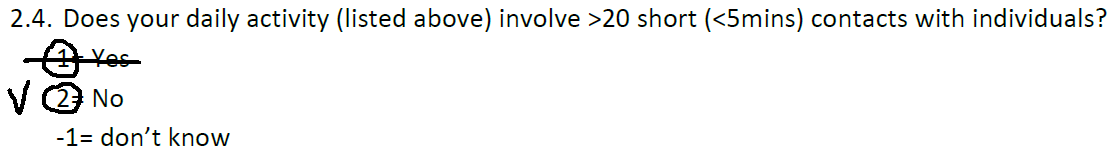


- Write the page number on the bottom right corner of each page of the questionnaire, as you go along
- For any numbers (e.g. participant’s age, dates), fill all boxes in. In case of a single digit number when a two-digit space is provided, write a zero in the first box (as shown below)


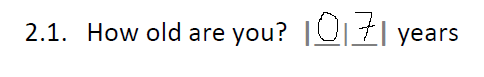


- During the interview you will ask the study participants to list their household contacts and their social encounters in the various settings where they spent their time during the previous day. As the number of such contacts and the number of places visited during a 24 hour period varies between individuals, the questionnaire comprises of tables (each row corresponding to one social contact in a particular setting) which can be extended if needs be just by adding extra pages to the questionnaire. Do not forget to add page numbers to the extra pages inserted.

1. **Part one: Socio-Demographic Characteristics**

- You will first explain the purpose of the survey and define what is meant by social contacts (including close physical and nonphysical contact and casual contact). This will have to be repeated during the interview when asking about the type of social encounters over a 24 hour period.
- You will then write the individual ID on the top left corner of each page of the questionnaire. The individual ID will also be used to label household (HH) contacts and other social contacts, where the first 4 digits correspond to the individual ID and the following digits to the contact-specific ID.
- The “surveyed day” refers to the day for which people are being asked to recall their social encounters. For example, if the survey takes place on a Monday at 10 am, the participants will be asked to recall their contacts between morning and evening of previous day (Sunday). The ‘surveyed day’ will thus be the Sunday.
- The first part includes questions about the individual’s daily activity, their household size and structure, and contact with animals. Please go through each question in the same order than on the questionnaire.
- Question 4 asks to list all household members and their age, sex and the relationship of the study participants with them (sibling, parent etc). It is advisable to list each household member’s name in the first column on the left hand side of the table, to make household contact identification easier later in the questionnaire.

1. **Part two: Settings**

- First explain to the participants what is meant by the previous day: from wake up to sleep.
- You will be asked to enter the number of the village/town visited based on a predefined list of settings **(see geographic area coding form)**
- Promptpeople to remember all the settings they went to by starting in the morning and then moving through their day to the evening.

1. **Part tree: Contacts**

- This is the longest part of the questionnaire. For each of the settings mentioned by the participants, you will be asked to record the number of social contacts and their characteristics.

- First start by telling the participant how social contacts are defined
- For each setting,**start by writing the names or the initials of the contacts,** then go back to each of the contacts mentioned and ask details about the characteristics of the contact (including type of contact, duration etc).
- The first setting will always be the household. As some of the social encounters are likely to take place with the same individuals in different settings, for each individual mentioned in the first setting (i.e. the household), you will ask whether contacts with the listed individuals have also taken place in other settings. If yes, you will already note the names or initials of such individuals in the tables recording the contacts in those other settings. This will also ensure that there is only ONE ID PER INDIVIDUAL, even if encounters with the same person take place in different settings. Similarly, as you progress through the interview, for each new contact (e.g. contacts in setting 2 that are not in setting 1), also ask whether contact was made in any other setting (e.g. 3, 4, 5 etc). Please see an example here below

- If the age of individuals is unknown, write down a best guess, with the letter A (i.e. **A**pproximation) before the age, as displayed below
- For each new setting, the contact ID number of new contacts (ie. not previously encountered in previously listed settings) will one number above the ID number of the last new contact mentioned in the previous setting. See example below
