# Supplementary material for: Characteristics of human encounters and social mixing patterns relevant to infectious diseases spread by close contact: a survey in Southwest Uganda
Source: BMC Infect Dis. 2018 Apr 11;18:172. doi: 10.1186/s12879-018-3073-1 (PMC5896105; doi:10.1186/s12879-018-3073-1)
Supplement: Supplementary file 2 — Supplementary Tables and Figures. (DOCX 1336 kb) [file 12879_2018_3073_MOESM2_ESM.docx]

**Additional File 2: Supporting Tables and Figures**

**TABLES**

Table S1: Age and Sex Distribution of Study Participants

| **Age category** | **Number (%) female** | **Number (%) male** | **Total number** |
| --- | --- | --- | --- |
| <2 years | 23 (38%) | 38 (62%) | 61 |
| 2 – 4 years | 26 (45%) | 31 (55%) | 57 |
| 5 – 9 years | 37 (50%) | 37 (50%) | 74 |
| 10 – 14 years | 22 (43%) | 29 (57%) | 51 |
| 15 - 24 years | 53 (58%) | 38 (42%) | 91 |
| 25 – 34 years | 45 (79%) | 12 (21%) | 57 |
| 35 – 44 years | 35 (64%) | 20 (36%) | 55 |
| 45 – 54 years | 35 (76%) | 11 (24%) | 46 |
| 55 – 64 years | 20 (77%) | 6 (23%) | 26 |
| 65+ years | 34 (71%) | 14 (29%) | 48 |

Table S2. Association Between Socio-demographic Variables and Level of Social Contacts. High social contact here is defined as ≥10 reported social contacts.

| **Variables** | **N** | **N (%) with high frequency of casual contacts (≥ 10)** | **Crude Risk Ratio (RR) (and 95%CI)** | **Adjusted RR (95%CI)** |
| --- | --- | --- | --- | --- |
| Age groups |  |  |  |  |
| <2y | 50 | 10 (20%) | 0.57 (0.30 ,1.09) |  |
| 2-4y | 47 | 14 (30%) | 0.85 (0.50 ,1.45) |  |
| 5-9y | 52 | 25 (48%) | 1.37 (0.86 ,2.20) |  |
| 10-14y | 43 | 19 (44%) | 1.26 (0.80 ,1.99) |  |
| 15-24y | 83 | 29 (35%) | ref |  |
| 25-34y | 53 | 14 (26%) | 0.76 (0.45 ,1.27) |  |
| 35-44y | 49 | 27 (55%) | 1.58 (1.11 ,2.25) |  |
| 45-54y | 43 | 20 (47%) | 1.33 (0.86 ,2.06) |  |
| 55-64y | 23 | 7 (30%) | 0.87 (0.42 ,1.80) |  |
| 65+y | 47 | 10 (21%) | 0.61 (0.32 ,1.14) |  |
| Sex |  |  |  |  |
| Female | 297 | 98 (33%) | ref |  |
| Male | 193 | 77 (40%) | 1.23 (0.96 ,1.59) |  |
| Occupation/daily activity |  |  |  |  |
| Pre-school child | 81 | 22 (27%) | ref | ref |
| Student | 132 | 53 (40%) | 1.22 (0.80 ,1.86) | 1.14 (0.74 ,1.74) |
| Office/Shop worker | 34 | 19 (56%) | 1.68 (1.11 ,2.54) | 1.41 (0.90 ,2.21) |
| Agriculture/Manual work | 132 | 47 (36%) | 1.07 (0.65 ,1.76) | 1.00 (0.61 ,1.62) |
| At home | 60 | 11 (18%) | 0.55 (0.30 ,1.00) | 0.51 (0.27 ,0.94) |
| Other | 51 | 23 (45%) | 1.38 (0.84 ,2.26) | 1.27 (0.77 ,2.08) |
| Day of the week |  |  |  |  |
| Weekday | 385 | 138 (36%) | ref |  |
| Sunday | 105 | 37 (35%) | 0.92 (0.67 ,1.26) | 1.04 (0.92 ,1.18) |
| Travel outside village/town in previous 24 hours |  |  |  |  |
| No | 374 | 118 (32%) | ref |  |
| Yes | 116 | 57 (49%) | 1.58 (1.23 ,2.04) | 1.54 (1.18 ,2.00) |

**FIGURES**

Figure S1: The Reported Duration of Contact By Age Group Among Study Participants, Sheema District, January – March 2014


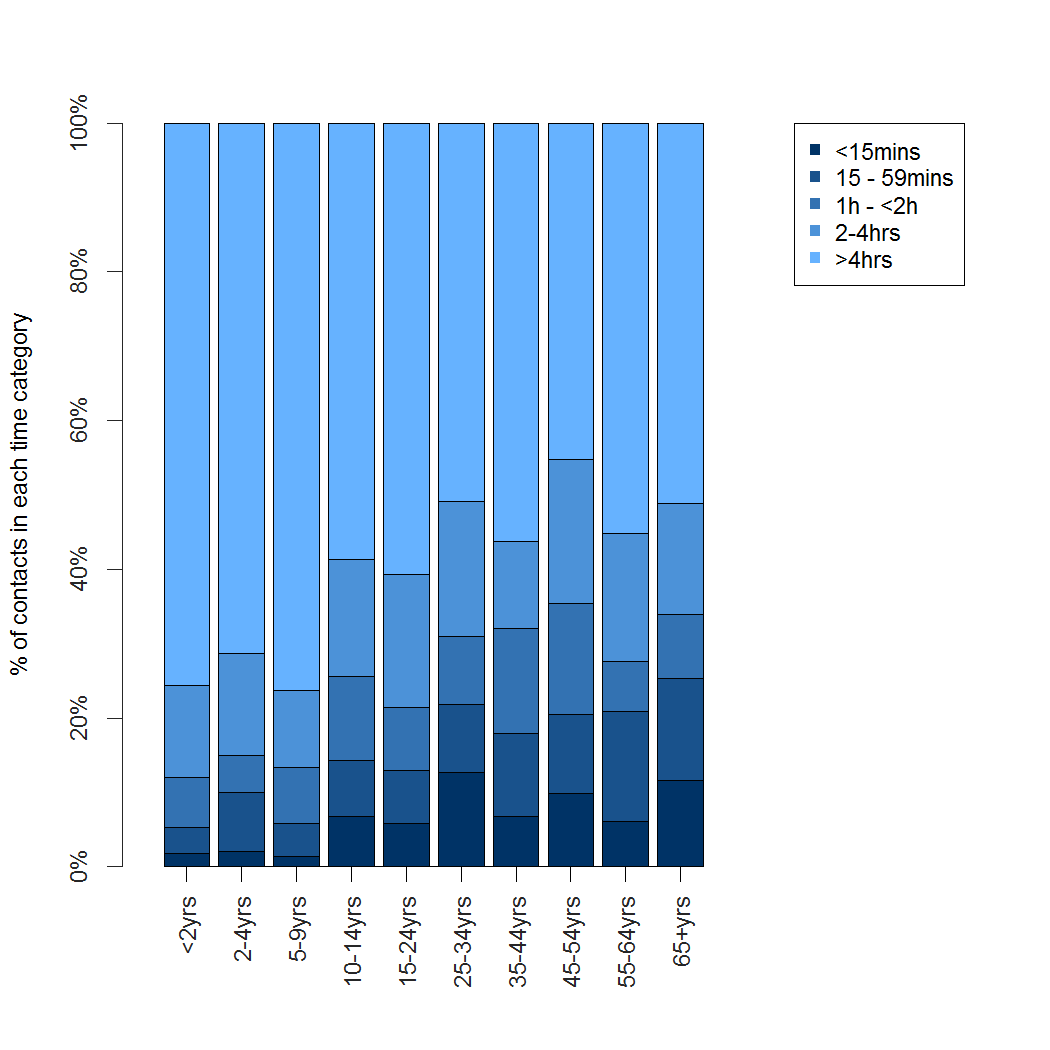


Figure S2a-c: Measure of assortativity of the contact matrices, Sheema District, January – March 2014. (S2A: median, S2B: lower 95% CI, S2C: upper 95%CI)


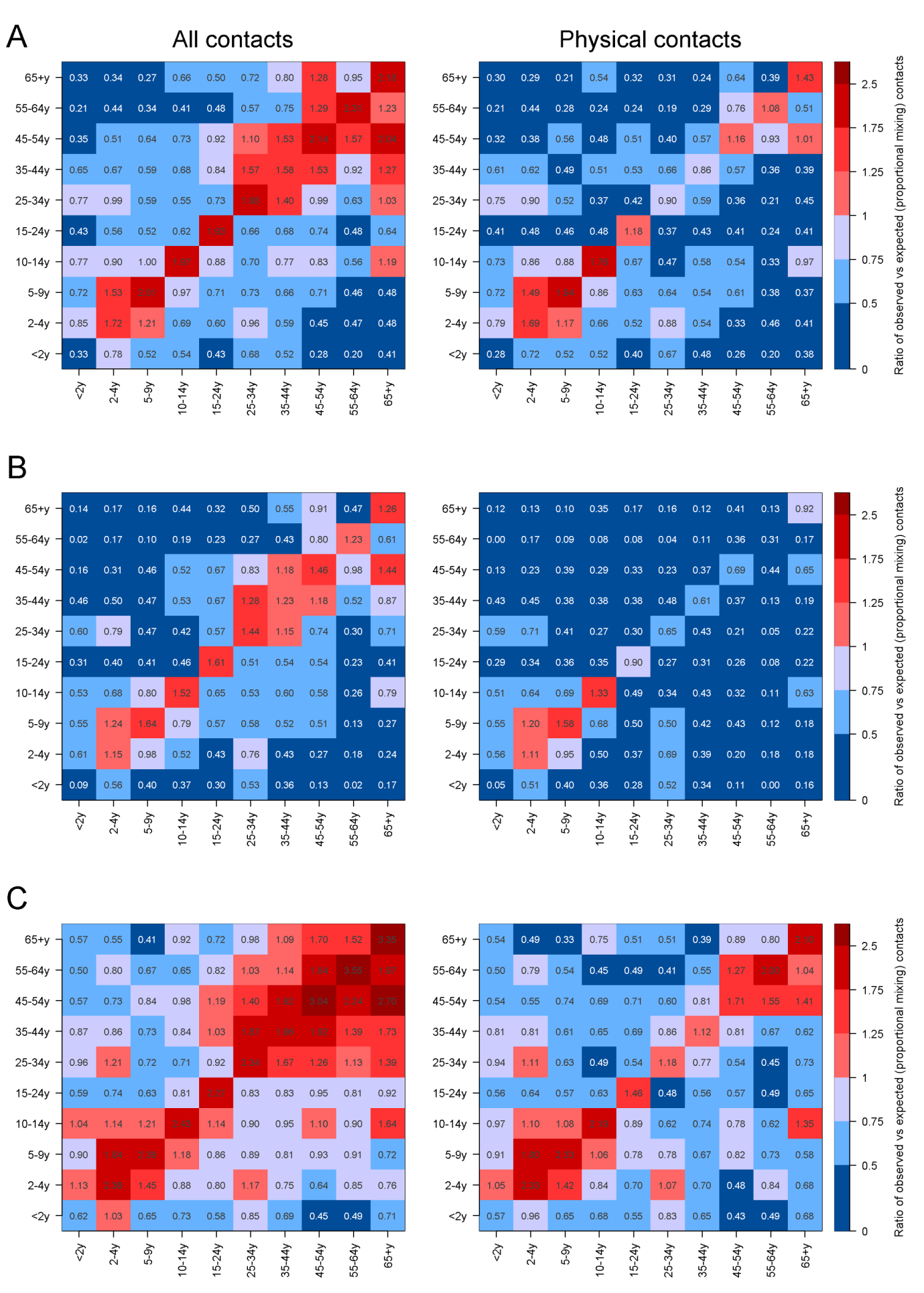


Legend: Left panel: all contacts; right panel: physical contacts. A: Median B:Lower 95%CI C:Upper 95%CI

Figure S3: Reciprocity Correction of the Contact Matrices for All Contacts, Sheema District, January – March 2014.


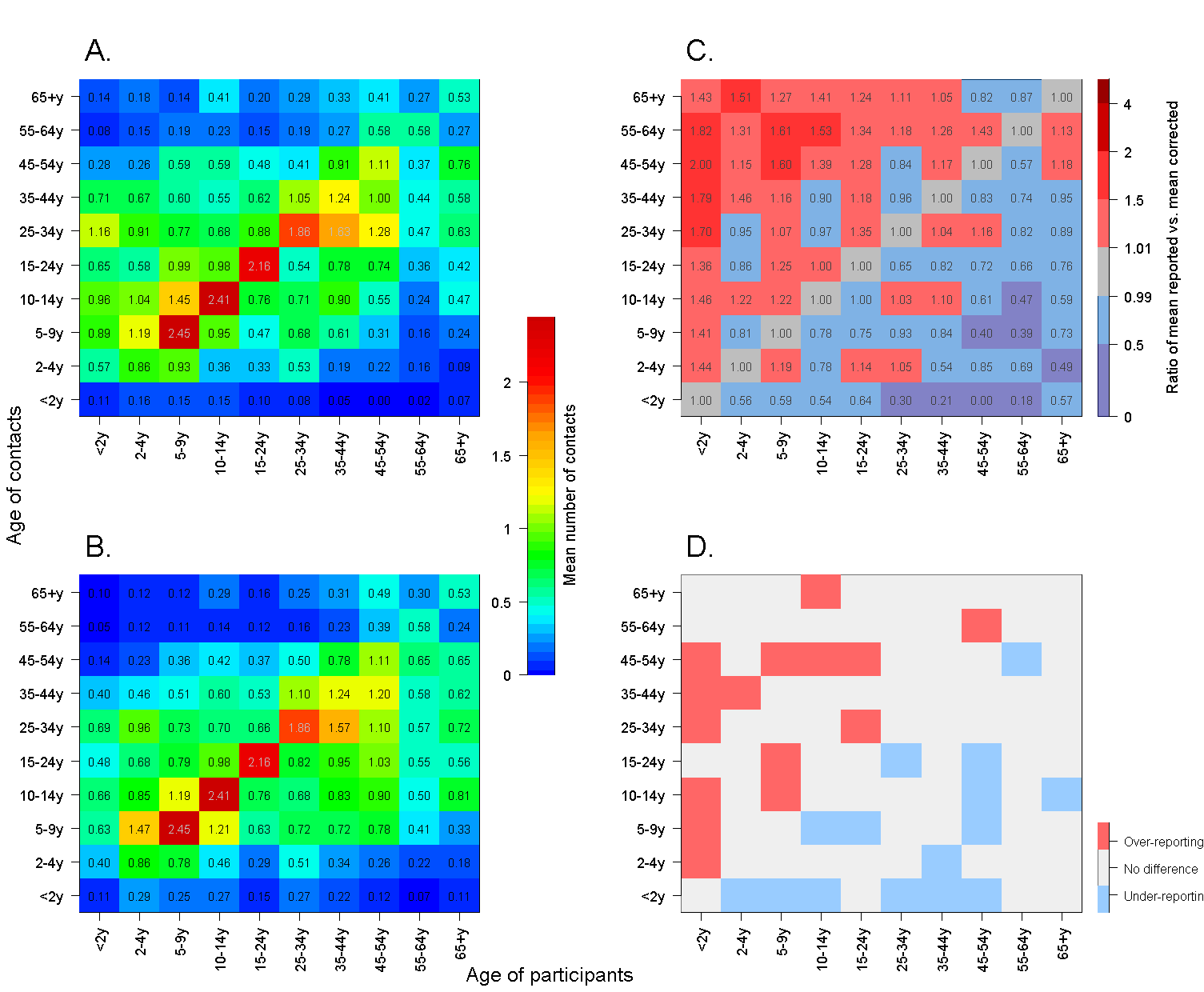


Legend: A) matrix for all reported contacts, not corrected. B) matrix for all reported contacts, corrected for reciprocity. C) Ratio of corrected over uncorrected matrices. Red cells illustrate where age-specific contacts were over-reported before correction, and blue cells under-reported. D) shows where participants significantly over-reported the number of age-specific contacts they had (upper 95% confidence bound) in red, significantly under-reported contacts in blue (lower 95% confidence bound), or where no significant adjustment was made (grey)

Figure S4: Epidemic Simulations Using Comparing Uganda and Great Britain, Assuming a 25% Underreporting Of Contacts in Uganda.


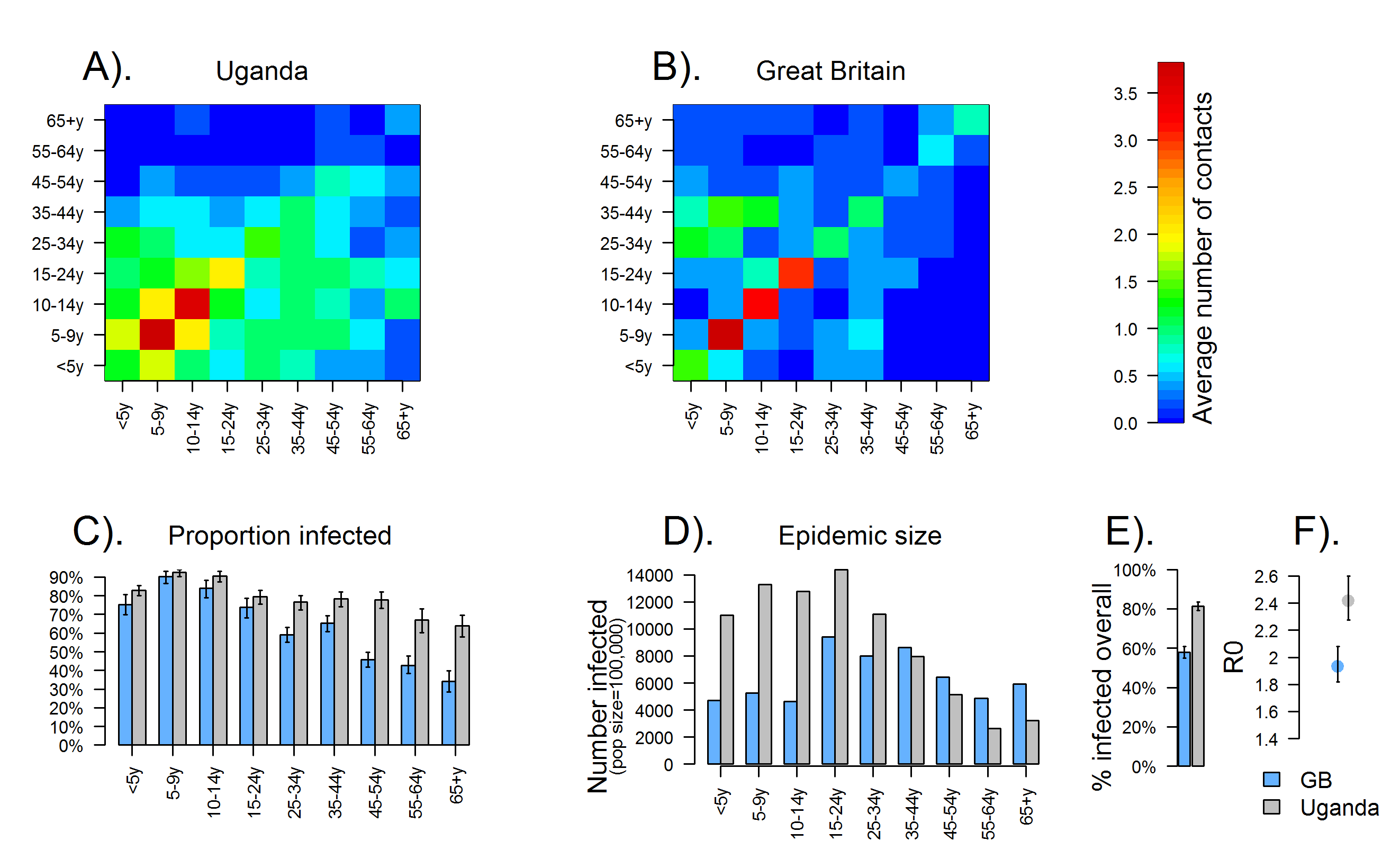


Legend: A) Physical Contacts from Uganda, B) Physical Contacts from Great Britain, C) Proportion Infected by Age Group, D) Epidemic Size by Age group, E) Overall Proportion Infected, and F) the Basic Reproduction Number R0.
