# Supplementary material for: Characteristics of human encounters and social mixing patterns relevant to infectious diseases spread by close contact: a survey in Southwest Uganda
Source: BMC Infect Dis. 2018 Apr 11;18:172. doi: 10.1186/s12879-018-3073-1 (PMC5896105; doi:10.1186/s12879-018-3073-1)
Supplement: Supplementary file 3 — Data dictionary. (DOCX 20 kb) [file 12879_2018_3073_MOESM3_ESM.docx]

**Additional File 3: Data dictionary**

The file labelled “data_contact_study.csv” contained the data used in this analysis, for 3,965 contacts, which all correspond to an individual row in the file. The definition of contact is anyone with whom a participant had a two-way conversational encounter lasting for ≥5 minutes, as described in the main paper (see Methods).

**Table S3** below provides a detail of each variable in the dataset

| **Variable** | **Explanation** |
| --- | --- |
| participant_id | Unique identification number for each participant |
| cluster_id | Unique identification number for each cluster from which individuals were sampled |
| agecat_participant | Age categories of the participant as used in the manuscript |
| occupation | Occupation of the study participant |
| day_surveyed | Week day preceding the survey day, about which information on contacts was asked as per definition (see Methods) |
| agecat_contact | Age categories of the contacts as used in the manuscript |
| frequency_of_interaction | Refers to how often the named contact is usually met by the participant. |
| relationship | Relationship of the contact with the study participant |
| contact_type | Physical (i.e. skin-to-skin touch) or non-physical |
| total_time_contact | Total time spent by the study participant with the named contact during the day preceding the survey day |
| number_casual_contacts | Estimates number of casual contacts (i.e. <5 minutes long) |
| distance_from_home | Provides the straight line distance (in km) of the centre point of the village/town of encounter from that of the village/town of residence of the study participant. Hence, all “0.0” reflect encounters within the village/town |
| Rural | Differentiates participants from rural villages (“Yes”) to those living in the two district towns (“No”) |
